# Supplementary material for: Learning epistatic gene interactions from perturbation screens
Source: PLoS One. 2021 Jul 13;16(7):e0254491. doi: 10.1371/journal.pone.0254491 (PMC8277066; doi:10.1371/journal.pone.0254491)
Supplement: S1 Appendix — (PDF) [file pone.0254491.s001.pdf]

A Fitness landscapes and epistasis

A.1 Conditional epistasis

For two genes i and j and a fixed set of background perturbations $b = (x_1, \ldots, x_{i-1}, x_{i+1}, \ldots, x_{j-1}, x_{j+1}, \ldots, x_p) \in \mathbb{R}_+^{p-2}$ we define conditional epistasis between gene i and j given b as

$$\begin{aligned}\varepsilon_{i,j|b} = f(&x_1, \ldots, x_{i-1}, 0, x_{i+1}, \ldots, x_{j-1}, 0, x_{j+1}, \ldots, x_n) \\ &+ f(x_1, \ldots, x_{i-1}, 1, x_{i+1}, \ldots, x_{j-1}, 1, x_{j+1}, \ldots, x_p) \\ &- f(x_1, \ldots, x_{i-1}, 1, x_{i+1}, \ldots, x_{j-1}, 0, x_{j+1}, \ldots, x_p) \\ &- f(x_1, \ldots, x_{i-1}, 0, x_{i+1}, \ldots, x_{j-1}, 1, x_{j+1}, \ldots, x_p) \quad (12)\end{aligned}$$

Proposition 1. *For the fitness landscape model (Equation 3), the interaction terms $\beta_{i,j}$ are independent of b and equal to conditional epistasis, that is, $\varepsilon_{i,j|b} = \beta_{i,j}$.*

Proof. Without loss of generality, we can consider $(i, j) = (1, 2)$. Let $b = (x_3, \ldots, x_p)$. In model (Equation 3) we have

$$\begin{aligned}\varepsilon_{1,2|b} &= f(0, 0, x_3, \ldots, x_p) + f(1, 1, x_3, \ldots, x_p) \quad (13) \\ &\quad -f(1, 0, x_3, \ldots, x_p) - f(0, 1, x_3, \ldots, x_p) \\ &= \beta_0 + \left(\beta_0 + \beta_1 + \beta_2 + \beta_{1,2} + \sum_{i>2} x_i\beta_{1,i} + \sum_{i>2} x_i\beta_{2,i}\right) \\ &\quad - \left(\beta_0 + \beta_1 + \sum_{i>2} x_i\beta_{1,i}\right) - \left(\beta_0 + \beta_2 + \sum_{i>2} x_i\beta_{2,i}\right)\end{aligned}$$

All terms except the interaction $\beta_{1,2}$ cancel out, therefore $\varepsilon_{1,2|b} = \beta_{1,2}$. □

A.1.1 Marginal epistasis

The marginal fitness landscape of genes i and j is

$$f_{i,j}(x_i, x_j) = \sum_{\{x_k \in \{0,1\} | k \neq i,j\}} f(x_1, \ldots, x_p) \quad (14)$$

and marginal epistasis between genes i and j is the epistasis of the marginal fitness landscape,

$$\varepsilon_{i,j} = f_{i,j}(0, 0) + f_{i,j}(1, 1) - f_{i,j}(1, 0) - f_{i,j}(0, 1) \quad (15)$$

For example, for $p = 3$ genes, marginal epistasis between gene 1 and 2 is

$$\begin{aligned}\varepsilon_{1,2} = [&f(0, 0, 0) + f(0, 0, 1)] + [f(1, 1, 0) + f(1, 1, 1)] \\ &- [f(1, 0, 0) + f(1, 0, 1)] - [f(0, 1, 0) + f(0, 1, 1)] \quad (16)\end{aligned}$$

Note that we consider the marginal epistasis of the *pair* of genes, rather than a single gene with all others as in [1])

Corollary 1. *For the fitness landscape model (Equation 3), the interaction terms* $\beta_{i,j}$ *are related to marginal epistasis via* $\varepsilon_{i,j} = 2^{p-2}\,\beta_{i,j}$.

(We use this definition of epistasis, rather than defining it in terms of averages, because it preserves $\beta_{i,j}$ as the value actually calculated by our model.)

Proof. From Proposition 1 we have that conditional epistasis for a pair of genes (i, j) and a fixed genetic background of the remaining $p-2$ genes equals $\beta_{i,j}$. There are 2^{p-2} such genetic backgrounds, and the conditional epistasis is the same for all of them. □

Thus, in the fitness landscape model (Equation 3), which contains all main effects and pairwise interactions, but no interactions of higher order, the interaction terms $\beta_{i,j}$ alone determine conditional and marginal epistasis of the fitness landscape.

B Number of epistatic gene pairs

For $n = 10 \times p = 1000$ siRNAs, 87% of the $\binom{p}{2} = 4950$ gene pairs are simultaneously perturbed by at least one siRNA.

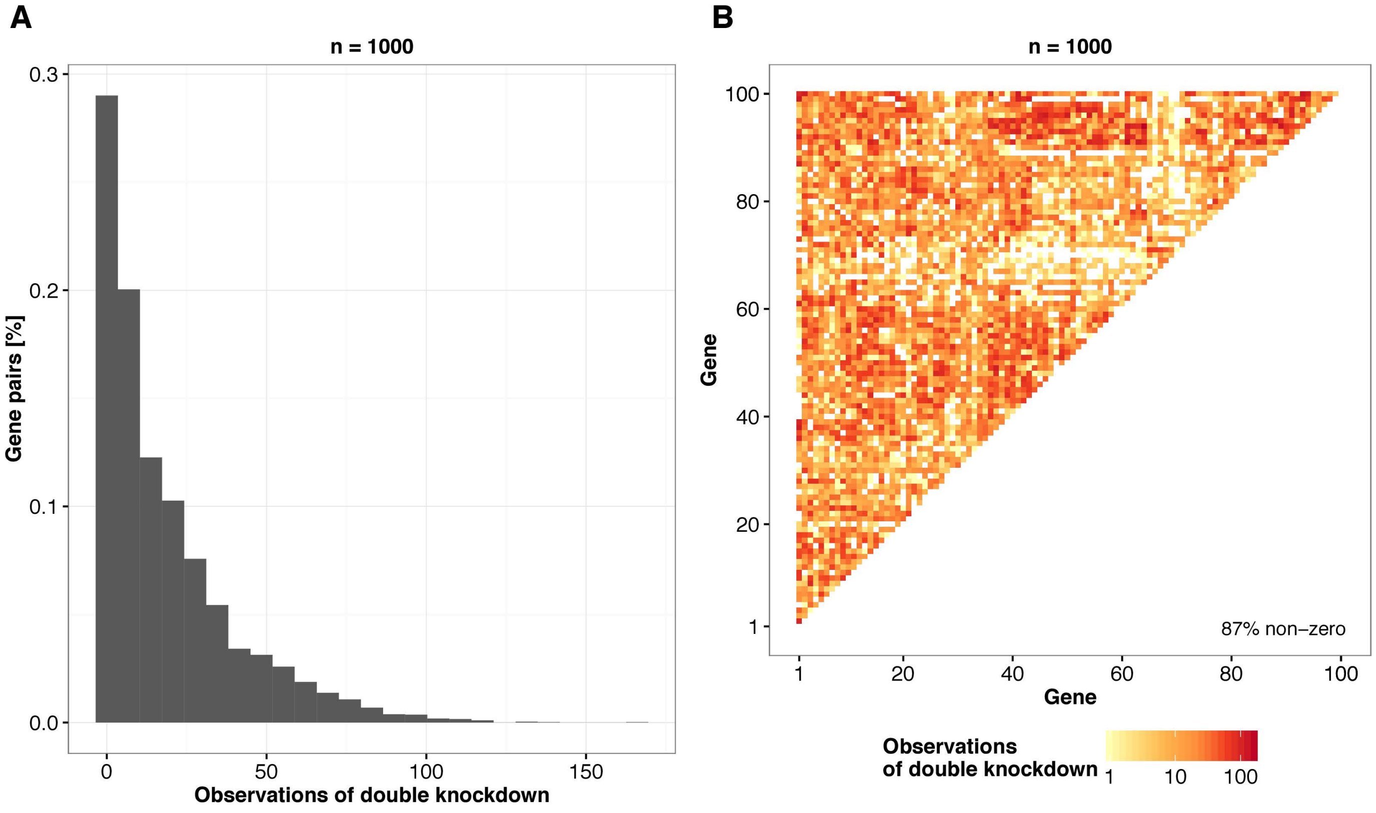

Fig 13. Simulation of perturbation matrices for $n = 1000$ siRNAs and $p = 100$ genes based on four commercial genome-wide siRNA libraries from Qiagen. A) The number of times each pair of genes is simultaneously perturbed in the simulated matrix. (**B**) Heat map of the number of simultaneous perturbations for each gene pair. Darker colour indicates higher numbers of observations. 87% of the $\binom{p}{2}$ pairs are simultaneously perturbed at least once.

An increase in the number of pairs of genes $(i, j) : \beta_{i,j} > 0$, i.e. pairs of genes with true conditional epistasis greater than zero, generally leads to an increase in precision and decrease in recall which results in a subtle increase in F1 when searching with `glinternet` (Fig. 14a). The only exception being when there are no additional main effects, in which case interactions are more reliably found from among a small set (between 5 and 20 depending on the SNR) than a large one (50 or more). When we select estimates $\hat{\beta}_{i,j}$ with a magnitude significantly different from zero (Fig. 14b), we observe a more than 3-fold increase of precision but steeper decrease of recall for increasing numbers of pairs of genes with true conditional epistasis. This results in approximately 2-fold increase of the F1 measure, which in addition shows a weaker dependency on the number of gene pairs with true conditional epistasis. With an increasing number of additional main effects, the performance generally decreases. The effect is more subtle for high numbers of true epistatic gene pairs, both with and without selecting $\hat{\beta}_{i,j}$ significantly different from zero. As expected, higher SNRs leads to better performance, where this effect is stronger when we perform the significance test. The trade-off between precision and recall resulting from the significance test is

shown in Fig. 2a. The increase in precision and decrease of recall is stronger for higher number of true epistatic gene pairs. For small numbers of true epistatic gene pairs (5, 20) we observe a dependency of the strength of increase of precision and decrease of recall to the number of additional main effects. Overall, the ratio of increase in precision and decrease of recall is approximately 3, suggesting that the test in general led to an increase in performance. Figures without this test may be found in Appendix E.

It should be noted that the expected precision of random guessing of interactions is $\frac{q}{p(p-1)}$. This is at most $\approx 1\%$, when $q = 100, p = 100$, as in our simulations.

(a) **(b)**

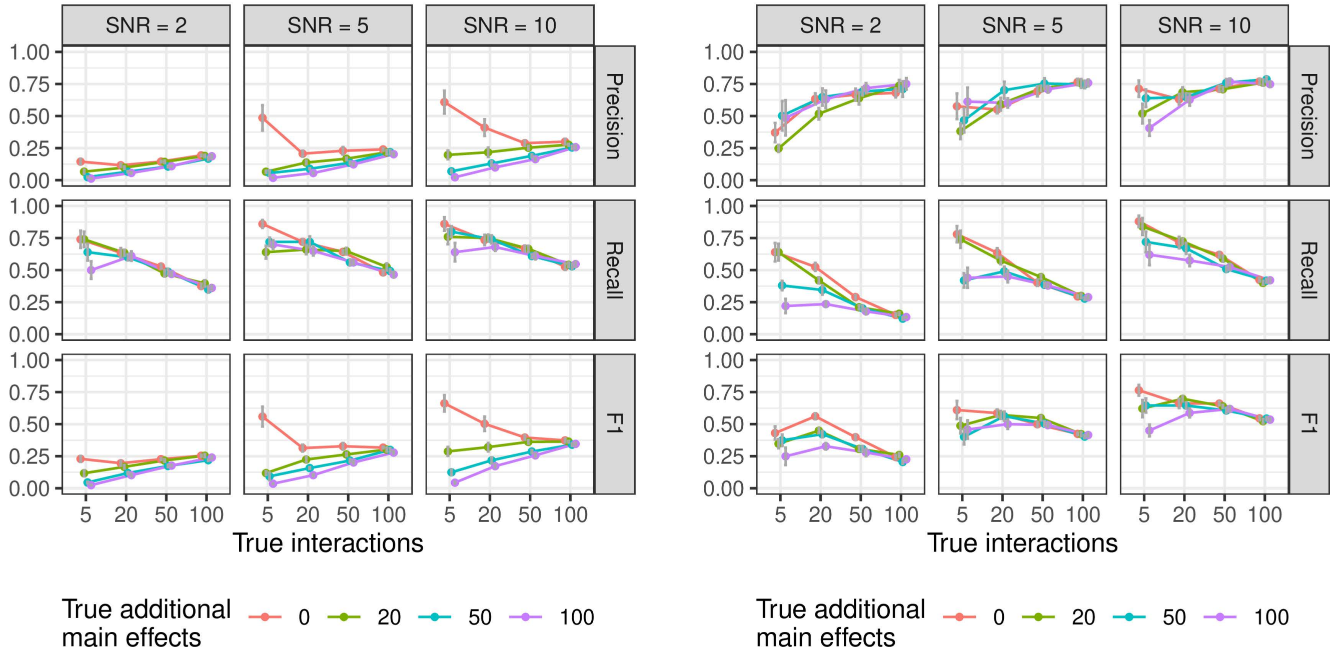

Fig 14. Identification of epistasis for increasing numbers of true interactions using `glinternet`. Panel rows show precision, recall, and the F1 measure and panel columns depict results for signal-to-noise ratios (SNR) 2, 5, and 10. Colour indicates the number of additional main effects not overlapping with the set of interacting genes. (a) Results for all conditional epistasis $\beta_{i,j} > 0$; (b) Results for the subset of conditional epistasis $\beta_{i,j}$ that significantly deviate from zero (q-value < 0.05).

Using the same number of genes and main effects and searching with `xyz`, we see similar precision, albeit with significantly lower recall (Fig. 15). As with `glinternet`, performance improved with higher SNRs. Selecting estimates that significantly deviate from zero (q-value < 0.05) results in as much as a 2-fold improvement in precision in the best case, however improvements are generally smaller with `xyz` than with `glinternet`. In this case, the effect on recall is minimal, the trade-off is shown in Fig. 2b.

(a) **(b)**

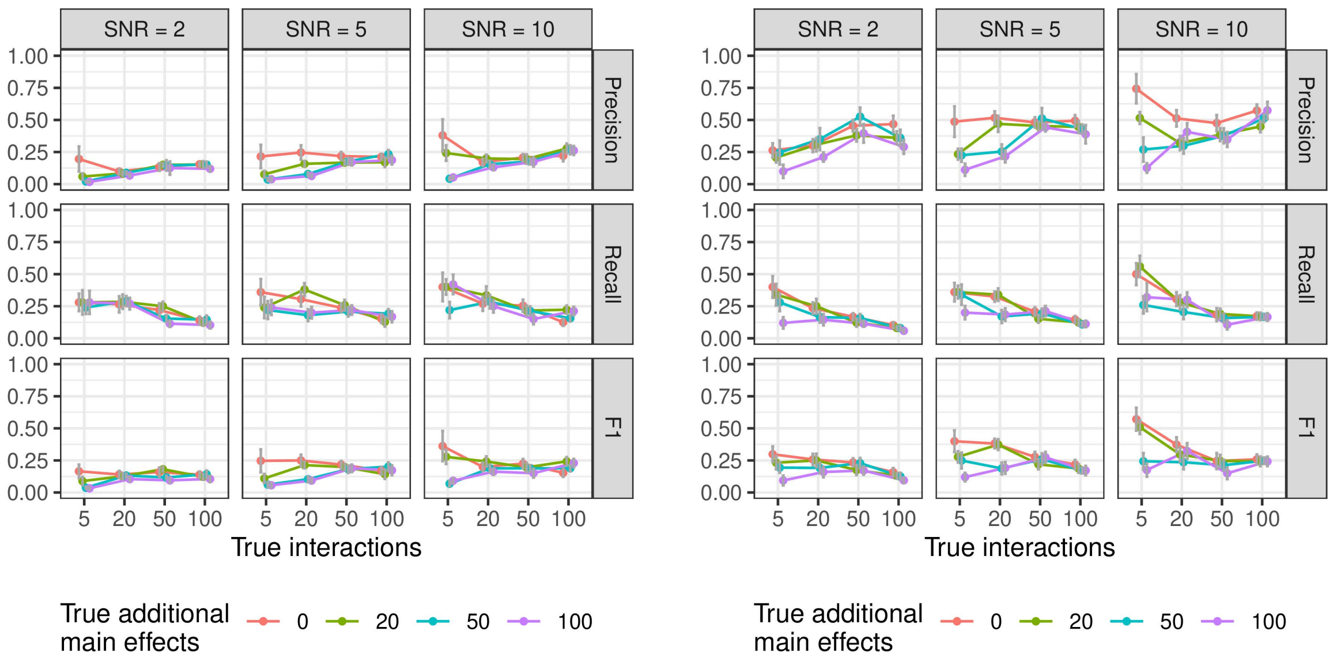

Fig 15. Results for `xyz` as in Fig. 14. Note that this format is reused in all such figures. (a) Without significance test. (b) With significance test.

C The `xyz` Algorithm

The xyz algorithm `xyz` defines $\mathbf{Z} \in \mathbb{R}^{n \times p} : Z_{i,j} = Y_i X_{i,j}$, and reduces the problem of finding pairs j, k such that $Y^T (X_j \circ X_k)/n > \lambda$ to that of finding j, k where $||X_j - Z_k||_2 < \lambda'$. The latter is achieved using locality sensitive hashing. Given parameters L, λ, the `xyz` algorithm is as follows:

Algorithm 1 The `xyz` algorithm

Require: L, λ

$I = \emptyset$
for L times **do**
    Choose a random vector $R \in \mathbb{R}^n$, and calculate $x = \mathbf{X}^T \mathbf{R}$ and $z = \mathbf{Z}^T \mathbf{R}$.
    The pairs j, k for which $x_j = z_k$ are candidates for interactions.
    Among these, add the pairs for which $|\mathbf{X}_j^T \mathbf{Z}_k|/n > \lambda$ to the set I.
end for
I is the set of significant Interactions, using the cut-off λ.

The parameter λ here is the same as the linear regression λ, and algorithm 1 can be

used during regression to choose the interaction effects worth considering at the current value of λ.

Unless otherwise specified, we use the value of $L = \sqrt(n)$. The effect of varying this parameter is considered in Appendix D.

D Number of xyz Projections

To ensure the correct `xyz` parameters are chosen, we compare precision, recall, and F1 for varying numbers of projections. Fixing the signal to noise ration to $SNR = 5$, and using the same parameters as the main $p = 1000$ simulations above, we run `xyz` with $L = 10, 100$, and 1000.

While there is a clear advantage to running at least $L = \sqrt(n) = 100$ projections, there are no significant gains in overall performance, as indicated by $F1$, beyond that. In fact, we can see in Fig. 16c that increasing the number of projections beyond that merely reduces the number of interactions returned, without improving accuracy.

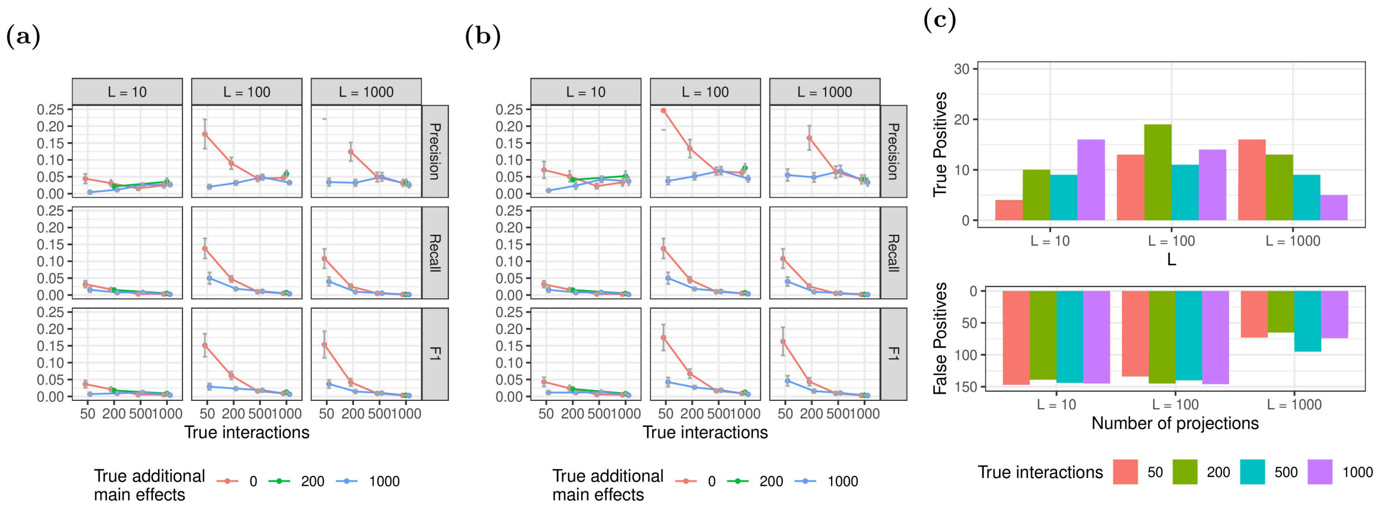

Fig 16. Precision, recall, and F1 as a result of increasing the number of projections. We use $p = 1000$ genes with a signal to noise ratio of five. (a) Results considering all identified conditional epistasis. (b) Results considering only the subset of conditional epistasis that significantly deviate from zero. (c) Number of interactions reported, note that the scale above differs from the one below for readability.

E Results without Significance Test

The results used above are using R's `lm` linear regression, including only those with significant q-values ($q < 0.05$). we perform the same tests with all found effects included, significant or otherwise, or comparison (Fig. 17 Fig. 18 Fig. 19 Fig. 20 Fig. 21 Fig. 22).

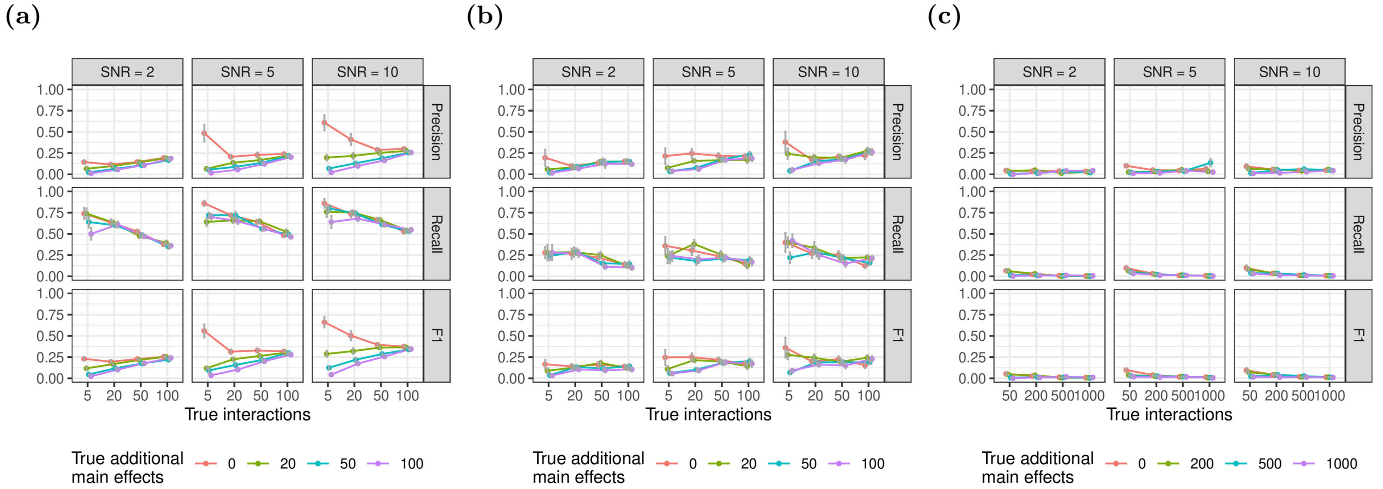

Fig 17. Precision, recall, and F1 performance measures for `xyz`. (a) Results for all identified epistasis on $p = 100$, $n = 100$ simulation using `glinternet`. (b) Results for all identified epistasis on $p = 100$, $n = 100$ simulation using `xyz`. (c) Results for all identified epistasis on $p = 1000$, $n = 10000$ simulation using `xyz`.

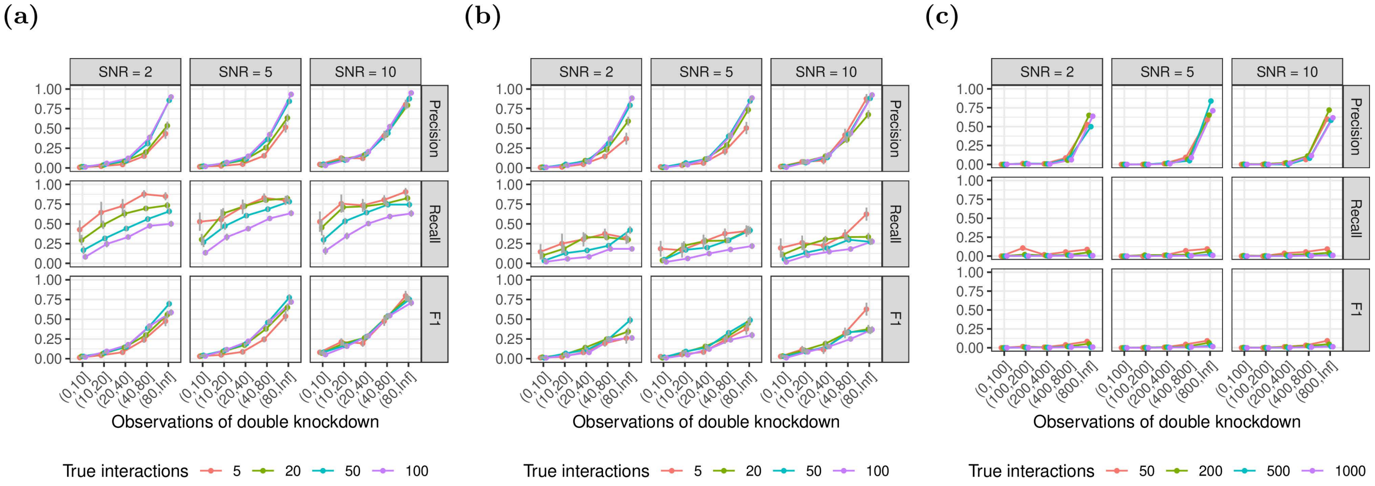

Fig 18. Identification of epistasis for increasing numbers of observations of the pairwise double knockdown. Results are for all identified conditional epistasis $\beta_{i,j} > 0$. (a) Results using `glinternet`. (b) Results using `xyz` on small $(p = 100, n = 1000)$ simulations. (c) Results using `xyz` on large $(p = 1000, n = 10000)$ simulations.

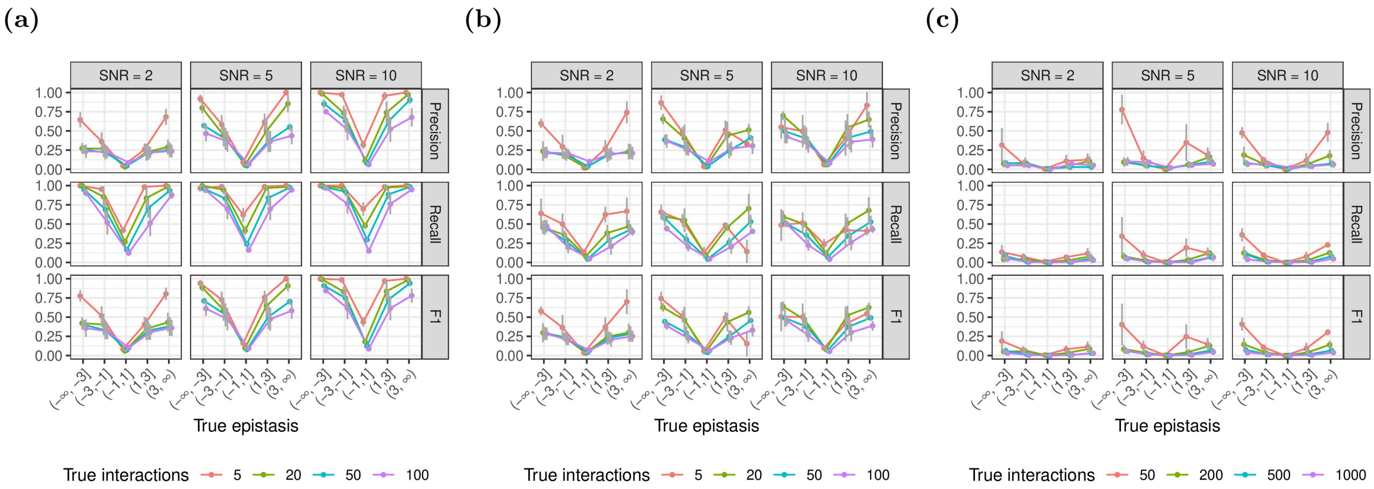

Fig 19. Identification of epistasis for varying effect size. Results are for all identified conditional epistasis $\beta_{i,j} > 0$. (b) Small $(p = 100, n = 1000)$ simulations, using `glinternet`. (b) Small $(p = 100, n = 1000)$ simulations, using `xyz`. (c) Large $p = 1000,\ n = 10000$ simulations, using `xyz`.

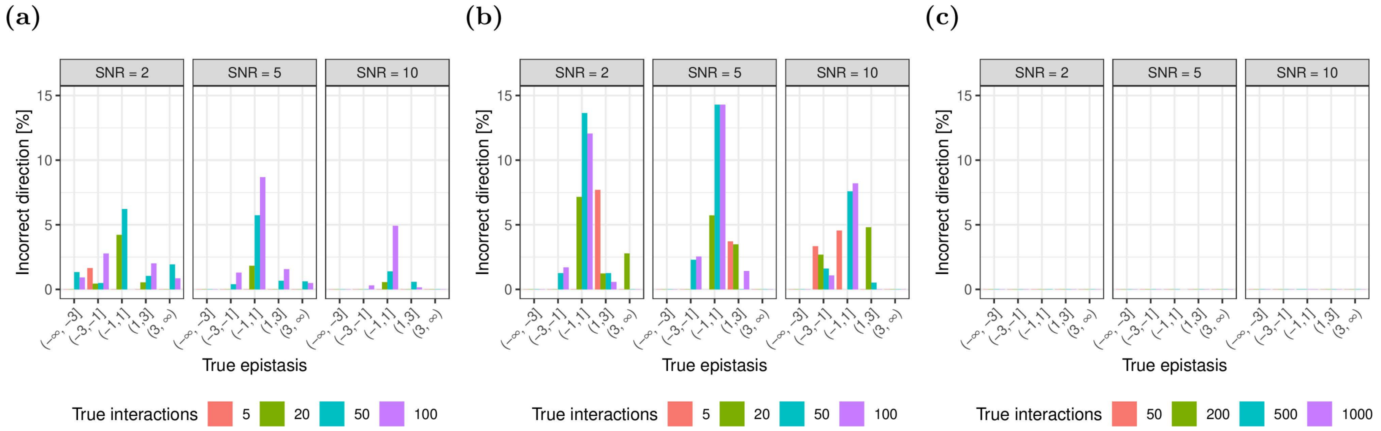

Fig 20. Identification of epistasis sign. Results are for all identified conditional epistasis $\beta_{i,j} > 0$. (b) Small $(p = 100, n = 1000)$ simulations, using `glinternet`. (b) Small $(p = 100, n = 1000)$ simulations, using `xyz`. (c) Large $p = 1000,\ n = 10000$ simulations, using `xyz`. Note that in this test there are no incorrect results

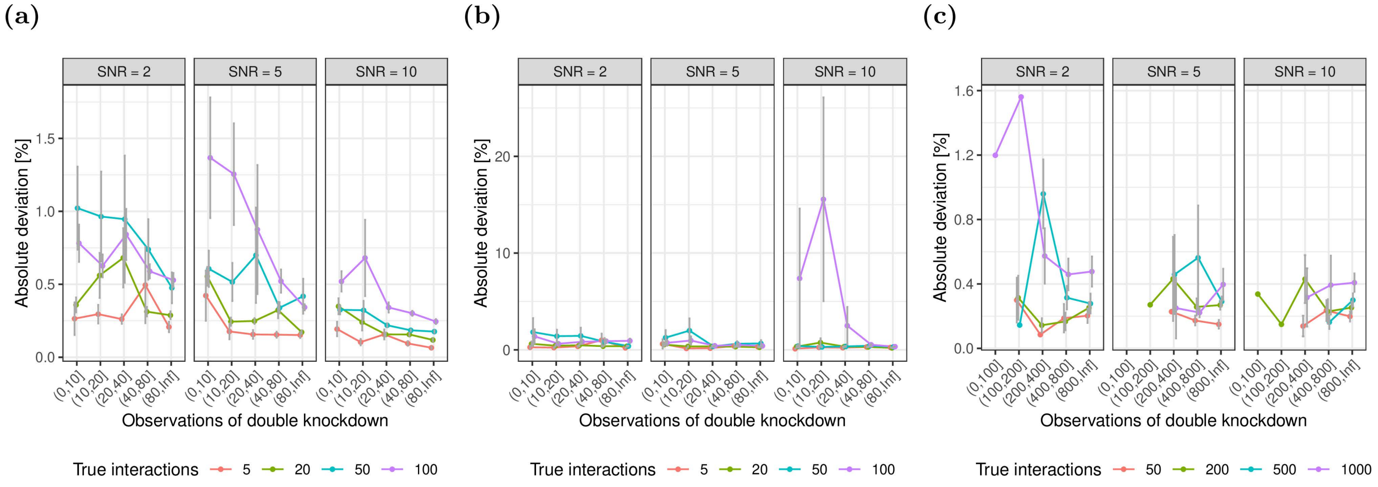

Fig 21. Concordance between the magnitude of true and estimated epistasis. Results are for all identified conditional epistasis $\beta_{i,j} > 0$. (a) Small ($p = 100, n = 1000$) simulations, using `glinternet`. (b) Small ($p = 100, n = 1000$) simulations, using `xyz`. (c) Large $p = 1000$, $n = 10000$ simulations, using `xyz`.

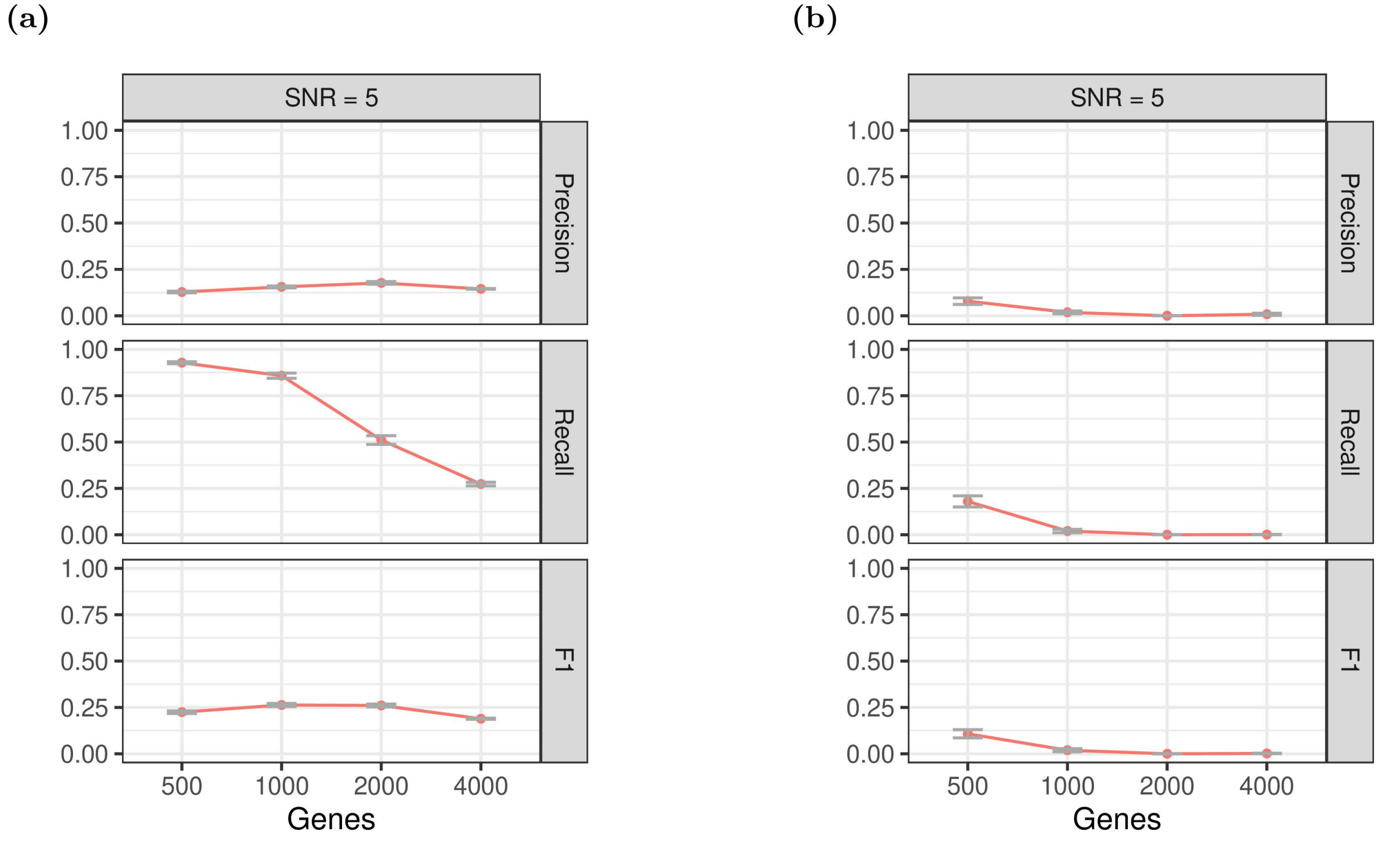

Fig 22. Performance on increasingly large data. (a) `glinternet` and (b) `xyz`.

Reference

1. Crawford L, Zeng P, Mukherjee S, Zhou X. Detecting Epistasis with the Marginal Epistasis Test in Genetic Mapping Studies of Quantitative Traits. PLOS Genetics. 2017;13(7):e1006869. doi:10.1371/journal.pgen.1006869.
