# Supplementary material for: Learning epistatic gene interactions from perturbation screens
Source: PLoS One. 2021 Jul 13;16(7):e0254491. doi: 10.1371/journal.pone.0254491 (PMC8277066; doi:10.1371/journal.pone.0254491)
Supplement: Attachment — Submitted filename: 31-03-2021-perturbations_response_to_plos_cb_referees.pdf [file pone.0254491.s002.pdf]

Response to Reviewers Comments

March 30, 2021

We would like to thank the reviewers for reading our manuscript and for their helpful suggestions. Many of these prompted changes in the paper, and we believe we have now addressed every point. We provide a point-by-point response to the reviewers comments below, and append the difference between the original and revised versions.

1 Reviewer 1

1. The discussion on setting up the epistasis model is cogent, easy-to-read, and certainly should not be removed, but it is also somewhat well-trodden territory that is somewhat secondary to the main points of the paper. Perhaps worth having a short overview and leaving the longer derivations to an appendix.

    - To make this section brief and to the point, we have moved the conditional and marginal epistasis proofs to the appendix.

2. A comparative weakness is any attempt to situate the paper within other efforts at imputing or measuring genetic interactions.

    - We have added in the introduction [paragraph 3] that we are not aware of any attempts to do so at the human exome scale. We therefore focus on demonstrating that this is a valid approach. We are not aware of any attempts to learn gene-gene interaction from siRNA perturbation screens at this scale either.

3. I overall found the article easy to read and largely self-contained. It is perhaps actually too long-winded at points.

    - It was our intention to make the article self-contained at the cost of the slightly increased length. Some proofs have, however, been moved into the appendix.

4. The idea of relying on off-target effects from RNAi to search for epistasis is new to me and intriguing. This is definitely the most novel aspect of the paper.

    - Thank you.

5. That said, a weakness is that remarkably little effort is then expended discussing the nature of RNAi off-target effects, which is a bit surprising given the long explanation of fitness epistasis. For one thing, my understanding is that many RNAi off-targets are driven by siRNAs functioning akin to microRNAs—broad but weak effects through partial complementarity. The original paper on off-targets (https://www.nature.com/articles/nbt831) suggests that most off-targets are on the order of two-fold changes, as opposed to 10-fold for direct targets. This doesn't quite fit the binary perturbation paradigm the authors impose.

- Predicting which off-target effects exist is an interesting and important subjects that is actively being studied [[2], [1]]. However, this is not a subject of our paper, so we, as explained, assume that the off-targets are given. All existing off-target prediction methods are for our purposes interchangeable. This assumption is important and results in the methods being applicable beyond siRNA perturbation screens. The binary perturbation vector only indicates whether effects exist, not their magnitude. The magnitude is determined by linear regression.

6. I'd also be interested to see discussion of the quality of off-target prediction, since it seems the usefulness of this approach is going to hinge critically on this. What happens if coefficients are misspecified?

    - The simulations with only a few main and interaction effects are also effectively those in which the off-target prediction is poor. The X matrix in these cases still contains many predicted simultaneous perturbations, they are just not considered 'real' effects for the simulation (i.e. they could be mis-predicted off-targets).
    - Furthermore, for our purposes off-target prediction methods are interchangeable, although we agree their quality is an important detail.

7. They bother to introduce the xyz search approach but spend most of their time in later experiments basically saying only glinternet is sufficiently accurate to be useful. Seems like a bit of an aside? Maybe better to put it in later in the context of scaling challenges and then say it has accuracy issues.

    - Given its excellent running time, a detailed look into where, if anywhere, xyz is usable seemed appropriate to us. In particular, there is some evidence that it can be used to find strong interactions, or those that occur frequently enough in the data, despite poor overall performance. These points have been clarified in the summary recommendation section.

8. Scoring a synthetic lethal as -1000 on a log scale is not a realistic assumption. Synthetic lethality does not have to mean instantaneous death to be useful – it is a quantitative phenotype. In most in vitro settings one would expect only partial phenotypes anyway due to the technical noise conferred by incomplete knockdown, cell-to-cell heterogeneity, etc.

    - True and this is intentional, as the rather extreme assumption here puts synthetic lethals as the best possible case for detection via regression. In practice the accuracy would likely be somewhere between this and that of a small negative effect, but this places an upper bound on the accuracy we can expect. This has been clarified in the text in section 2.3.

9. A lot of time is spent on experiments with completely simulated data. Given that there are large empirical GI maps that have been measured in human cells, it seems like a major missed opportunity to not at least try to cross-reference results. (E.g. https://www.cell.com/cell/pdf/S0092-8674(18)30735-9.pdf, things it cites, things that cite it.) Some of these have been done with CRISPR and so in a sense provide a gold standard reference where you could look in RNAi studies and see if there are signs of the related interactions. As it stands the recommendations of when the approach might work are impossible to apply because they are phrased relative to arbitrary model parameters of SNR and effect size. There is a throwaway reference to

Costanzo et al. saying that it is challenging to compare to? Why is it challenging? Given that these are real data they have to be the standard against which a method or model is compared.

- A more general end-to-end attempt to consider accuracy of interactions in real data is present in section 2.6, and we cross-reference the predicted interactions with the string database [3]. This database includes known interactions from a variety of sources, including experimentally verified interactions.

  More generally, our goal is to establish the validity of using regularised regression to fit such perturbation screens, on the assumption they are accurate. If results in real data do not reflect known interactions it is impossible to say whether or not the perturbation and regression steps are at fault, the off-target predictions, or the interactions are simply not known. A more detailed look at this larger process would indeed be worthwhile, but is beyond the scope of our goals here.

10. The discussions on scalability are important but I have two major reservations here. First, frankly in 2020 it is not reasonable to say that you tried 10 cores and ran out of steam. If you can't use an HPC cluster, just use AWS. Second, this points to a more serious question of whether the proposed methods are actually practical. The point of RNAi and other genome-scale approaches is to work at genome-scale. If the computational methods don't extend to that scale, then a rigorous procedure for how to reduce scale should at least be discussed; the scaling dilemma and how to overcome it is essentially the central challenge of studying interactions.

    - The scalability of glinternet is limited by Ahmdal's Law. With 10 cores the majority of the running time is spent on single-threaded tasks, and any further gains would be negligible. It is the fundamental limit of the method to scale to larger data sets, rather than computational resources, that we wanted to demonstrate here. Our results show that no matter how many cores are employed, glinternet would not be able to learn interaction beyond the scale that we have identified. We have clarified this limit in section 2.2.

11. How are p-values assigned to significant interactions?

    - P-values are calculated using a t-test with $n - k - 1$ degrees of freedom to determine whether the estimated effect strength significantly deviates from zero. This has been clarified in section 1.2 of the text.

2 Reviewer 2

2.1 Main Comments

This paper describes a technique for inferring epistatic interactions from siRNA perturbation screens. The main ideas are (1) to treat the off-target effects of each siRNA together with the on-target effects as a complex perturbation of many genes and then (2) to extract epistatic interactions from a collection of these complex perturbations (i.e. a typical siRNA screen) using a sparse regression framework. The paper sets up this framework, conducts an extensive simulation study

where this approach is implemented using two different existing methods for sparse (elastic net) regression, and provides a reanalysis of an existing siRNA dataset.

My overall assessment is that the basic strategy described here is interesting, creative, and well-founded but that as it stands the manuscript is at the low end of the level of significance typical of PLoS Comp Bio papers. The main issue is that the bulk of the paper is dedicated to a detailed simulation study that would only be of interest to a very limited audience. At the same time, the analysis of real biological data is very short and what is presented suggests that the interactions found are plausible but does not really validate these predictions. I suggest that this paper could be revised to be suitable for publication at PLoS Comp Bio by providing more applications to real data (e.g. to another dataset or comparing with another method for inferring epistatic interactions, or applying it in a situation where gold-standard epistatic pairs are already known) and moving some of the simulation study to the SI.

- While a detailed analysis of real data, including verifying predictions, is undoubtedly worthwhile, we only aimed to show that the perturbation screen to regression step is valid. Testing the method on real data is implicitly testing a sequence of steps: off-target prediction, our perturbation screen regression, and the accuracy of the 'known' results. We do not claim that current off-target prediction methods are accurate, and determining this is beyond the scope if this paper. This has also been discussed in our responses to comments 9,6 and 5 from Reviewer 1, and a number of corresponding changes have been made in the text.

2.2 Additional Comments

1. I think it is important to more explicitly address the problem of how the specific patterns of off-target effects influence the number/fraction of true pairwise interactions. The authors note in the simulation section that interactions between gene pairs that are not simultaneously perturbed in the siRNA library are not structurally identifiable, but my intuition is that this structural non-identifiable subset could often constitute a large fraction of possible interactions. This suggests that analyses for different siRNA libraries might be expected to produce top hits that are indeed true epistatic interactions, but which would hardly overlap between studies using different libraries.

    - Any pair of genes can be perturbed in the siRNA screen by combining siRNAs that are known to target both. There is, therefore, no fundamental limit imposed by the structure of off-target effects. More importantly, we found over 80% of possible pairs were simultaneously perturbed at least once in our simulations, which were sample from a real off-target prediction matrix. Some of these pairs may not be distinguishable if they are perturbed exactly when another pair also is, but even a single difference would lead to one being preferred over the other as an explanatory effect. Figure 10 shows that only 13% of pairs are not perturbed in our simulations, and we note that the ratio is similar for the larger simulation. This is also mentioned, and has been clarified, in the text in section 2.1.

2. The definition of marginal epistasis could be improved by defining it in terms of averages rather than a sums. Then the fitness of a genotype in the marginal fitness landscape would simply be the expected fitness of a genotype given its allelic state at the specified subset of genes, and Corollary 1 simplifies to $\epsilon_{i,j} = \beta_{i,j}$ instead of $\epsilon_{i,j} = 2^{p-2}\beta_{i,j}$.

- This simplifies the definition of epistasis, but the β_{i_j} values given are the parameters our model actually estimates. This is purely here to show that this is equivalent to estimating epistasis. We have clarified this point in a footnote on page 24.

3. The authors should also disambiguate between their concept of marginal epistasis and the (different) marginal epistasis concept studied in e.g. Crawford et al. 2017 PLoS Genetics.

   - Crawford et al. consider marginal effects of a single gene, capturing all interactions with this one. This is fundamentally different to the marginal epistasis of a *pair* of genes. Nonetheless, we clarify this distinction in the Appendix A.

4. Sparse regression (Lasso, elastic map, and their Bayesian analogs) has previously been applied both in the fitness landscape and the quantitative genetics literature, and the reader should be aware of how the current contribution fits into this broader context. I am thinking of things like Cai et al. 2011 "Fast empirical Bayesian LASSO for multiple quantitative trait locus mapping" in the quantitative genetics literature and Otwinowski and Nemenman 2013 and Poelwijk et al. 2019 in the fitness landscape literature, but there is additional work in both areas (especially in quantitative genetics). One important feature of L1 regularized regression is that the results are sensitive to the choice of basis, and the Taylor basis used here is one difference with most other treatments of epistasis via regularized regression.

   - Poelwijk et al. 2019 and Nemenman 2013 study near complete measurements of very small genotype spaces (on the order of dozens of genes). This is important for understanding epistasis in general, but inapplicable to human genome scale data. We have added these to the discussion of smaller-scale fitness landscapes in the introduction.
   - The Bayesian Lasso method from Cai et al. 2011 improves accuracy over glmnet, but not running time. Glmnet is already far too slow for human genome-scale interactions, so this method is also not suitable for our purposes.

5. Although I suggest less emphasis on the simulation study, I do think it is necessary to say somewhat more about how the xyz algorithm works in order to understand the manuscript, particularly concerning questions about the number of projections used.

   - We are specifying the parameters to ensure reproducibility of our results. We have added a description of the xyz algorithm itself as appendix D. This is kept as an appendix since the discussion of xyz is seen as largely irrelevant by Reviewer 1 (comment 7).

6. The re-analysis of the siRNA screen should be clarified. The current text says that the kinases being targeted are kinases of the pathogens, but it appears that these are in fact the kinases of the human host. More generally, the reader needs more background on the experimental design of this study in order to understand the reanalysis. In Table 1, the reader also needs to see the corresponding single mutation effects in order to understand what kind of epistatic interactions these are (e.g. which of these are synthetic lethals), and some of the estimated effects seem awfully large (e.g. 172.04 on a log 2 scale).

   - The estimate of the effect strength is certainly suspect. The majority of the paper is focused on whether the estimated effects are actually present, and in the correct direction. A real screen then gives us a list of possible effects, a significant fraction of which may be there and if so are likely to be positive/negative as specified. To understand the exact effect of particular interactions we would then suggest a more fine-tuned investigation.

- We have added the main effect predictions to Table 1, as well as a short discussion of which effects may, in fact, be synthetic lethals.
- The pathogens were not the target, this was a mistake and has been corrected.

References

[1] Ferhat Alkan et al. "RIsearch2: Suffix Array-Based Large-Scale Prediction of RNA–RNA Interactions and siRNA off-Targets". In: *Nucleic Acids Research* 45.8 (May 5, 2017), e60–e60. ISSN: 0305-1048. DOI: `10.1093/nar/gkw1325`. URL: `https://academic.oup.com/nar/article/45/8/e60/2929519` (visited on 11/17/2020).

[2] Stefanie Lück et al. "siRNA-Finder (Si-Fi) Software for RNAi-Target Design and Off-Target Prediction". In: *Frontiers in Plant Science* 10 (2019). ISSN: 1664-462X. DOI: `10.3389/fpls.2019.01023`. URL: `https://www.frontiersin.org/articles/10.3389/fpls.2019.01023/full?utm_source=S-TWT&utm_medium=SNET&utm_campaign=ECO_FPLS_XXXXXXXX_auto-dlvrit` (visited on 03/29/2021).

[3] *STRING: Functional Protein Association Networks.* URL: `https://string-db.org/cgi/about.pl` (visited on 07/22/2020).

LEARNING EPISTATIC GENE INTERACTIONS FROM PERTURBATION SCREENS

KIERAN ELMES[1], FABIAN SCHMICH[2,3], EWA SZCZUREK[4], JEREMY JENKINS[5], NIKO BEERENWINKEL[2,3,✉], AND ALEX GAVRYUSHKIN[1,✉]

Abstract. The treatment of complex diseases often relies on combinatorial therapy, a strategy where drugs are used to target multiple genes simultaneously. Promising candidate genes for combinatorial perturbation often constitute epistatic genes, i.e., genes which contribute to a phenotype in a non-linear fashion. Experimental identification of the full landscape of genetic interactions by perturbing all gene combinations is prohibitive due to the exponential growth of testable hypotheses. Here we present a model for the inference of pairwise epistatic, including synthetic lethal, gene interactions from siRNA-based perturbation screens. The model exploits the combinatorial nature of siRNA-based screens resulting from the high numbers of sequence-dependent off-target effects, where each siRNA apart from its intended target knocks down hundreds of additional genes. We show that conditional and marginal epistasis can be estimated as interaction coefficients of regression models on perturbation data. We compare two methods, namely `glinternet` and `xyz`, for selecting non-zero effects in high dimensions as components of the model, and make recommendations for the appropriate use of each. For data simulated from real RNAi screening libraries, we show that `glinternet` successfully identifies epistatic gene pairs with high accuracy across a wide range of relevant parameters for the signal-to-noise ratio of observed phenotypes, the effect size of epistasis and the number of observations per double knockdown. `xyz` is also able to identify interactions from lower dimensional data sets (fewer genes), but is less accurate for many dimensions. Higher accuracy of `glinternet`, however, comes at the cost of longer running time compared to `xyz`. The general model is widely applicable and allows mining the wealth of publicly available RNAi screening data for the estimation of epistatic interactions between genes. As a proof of concept, we apply the model to search for interactions, and potential targets for treatment, among previously published sets of siRNA perturbation screens on various pathogens. The identified interactions include both known epistatic interactions as well as novel findings.

[1] Department of Computer Science, University of Otago, Dunedin, New Zealand
[2] Department of Biosystems Science and Engineering, ETH Zurich, Basel, Switzerland
[3] SIB Swiss Institute of Bioinformatics, Basel, Switzerland
[4] Institute of Informatics, University of Warsaw, Warsaw, Poland
[5] Novartis Institutes for BioMedical Research, Cambridge, Massachusetts, United States
E-mail addresses: `kelmes@cs.otago.ac.nz`, ✉`niko.beerenwinkel@bsse.ethz.ch`, ✉`alex@biods.org`.

Introduction

Genetic interactions are also referred to as epistasis, a term that originates from the field of statistical genetics and describes genetic contributions to the phenotype that are not linear in the effects of single genes (Wright 1932; Cordell 2002). Considering two genes at a time, positive and negative epistasis refer to a greater and smaller effect, respectively, of the double mutant genotype than expected from the two single mutant genotypes relative to the wild type. In genetics, the phenotype of primary interest is the reproductive success of a cell, which is commonly termed fitness (Orr 2009). In this context, a fitness landscape is the mapping of each combination of possible configurations of gene mutations to a fitness phenotype (de Visser, Cooper, and Elena 2011).

The knowledge of fitness landscapes is highly relevant for personalized disease treatment (Kaelin 2005). In cancer, for example, genetic aberrations result in cells with increased somatic fitness, for instance, by evading apoptosis or gaining the ability to metastasise. This increase subsequently promotes post-metastatic tumour development (Hanahan and Weinberg 2011). A major challenge in cancer therapy is the fact that many genes with driving mutations cannot be adequately targeted for inhibition due to toxic side effects and rapid development of drug resistance (Force and Kolaja 2011; Holohan et al. 2013). To overcome this challenge, a strategy based on the inhibition of genes that interact with genes with cancer driving alterations was proposed (Ashworth, Lord, and Reis-Filho 2011). This strategy is based on the principle of synthetic lethality (Kaelin 2005; Jerby-Arnon et al. 2014; O'Neil, Bailey, and Hieter 2017), the extreme case of negative epistasis, where single mutants are compatible with cell viability but the double mutant results in cell death. Identifying synthetic lethal gene interactions allows targeting cancer cells in which one of the two genes is mutated, by using drugs that affect the other. In the presence of this drug, the cancer cell lineage will no longer be viable (Chan and Giaccia 2011).

The identification of fitness landscapes is however a very challenging task, simply due to the exponential growth of the space of interactions. For yeast, for example, it has been shown to be feasible to experimentally perform 75% of all pairwise knockouts (Costanzo et al. 2010). Similarly, (Poelwijk, Socolich, and Ranganathan 2019; Otwinowski and Nemenman 2013) study fitness landscapes with a small number of genes in which all or nearly all genotypes of interest have been measured. However, in humans, with approximately 20,000 protein-coding genes, this would constitute to almost 200 million experiments to test all pairwise interactions. An approach that has been successfully applied to identify synthetic lethality *in vitro* is large-scale perturbation screening of human cancer cell lines using RNA interference (Steckel et al. 2012; Laufer et al. 2013; McDonald et al. 2017; DepMap 2020). However, this strategy only allows cataloguing synthetic lethal gene pairs where one gene is always specific to the screened cell line. While these methods may be sufficient for the identification of a few promising targets for cancer therapy, they do not allow us to estimate general pairwise gene interactions at the human

exome scale. To our knowledge, there are currently no methods for inferring gene interactions at this scale. We therefore focus on demonstrating that our approach is sound, rather than comparing to existing methods.

Short-interfering RNAs (siRNAs), the reagents used in RNAi perturbation screening, exhibit strong off-target effects, which results in high numbers of false positives rendering the perturbations hard to interpret (Jackson et al. 2006). While this is usually conceived as a problem, here we take advantage of this property for the estimation of genetic interactions (Schmich et al. 2015; Srivatsa et al. 2018; Tiuryn and Szczurek 2019). We propose a novel approach for the second order approximation of a human fitness landscape by inferring the fitness of single gene perturbations and their pairwise interactions from RNAi screening data (Figure 1). Our approach is not restricted to interactions with mutant genes of a specific cell line or explicit double knockdowns. We leverage the combinatorial nature of sequence-dependent off-target effects of siRNAs, where each siRNA in addition to the intended on-target knocks down hundreds of additional genes simultaneously. Not distinguishing between on- and off-targeted genes, we consider each siRNA knockdown as a combinatorial knockdown of multiple genes. Hence, every large-scale RNAi screen, though unintended, contains large numbers of observations of high-order combinatorial knockdowns and provides a rich source for the extraction of pairwise epistasis. These off-target effects have previously been used to improve inference of signalling pathways among a small number (on the order of a dozen) genes (Srivatsa et al. 2018; Tiuryn and Szczurek 2019). Here, however, we attempt to use it to discover epistatic gene pairs in a genome-wide fashion (i.e. among tens of thousands of genes). Our approach is formulated as a regularized regression model. It can also be deployed for the estimation of epistasis from phenotypes other than fitness, such as for instance phenotypes that measure the activity of disease-relevant pathways, e.g. for pathogen entry (Rämö et al. 2014), TGFβ-signalling (Schultz et al. 2011), or WNT-signalling (Tang et al. 2008). Long term, the identification of disease-relevant epistatic gene pairs may allow the design or re-purposing of agents for combinatorial therapy with the potential to improve the efficacy of drugs.

In solving this model, we adapt two recent statistical learning methods, namely `glinternet` (Lim and Hastie 2015) and `xyz` (Thanei, Meinshausen, and Shah 2018) to select genes and gene-pairs with non-zero effects on fitness, and evaluate both models on simulated data from real RNAi libraries. We vary the signal-to-noise ratios, number of true gene–gene interactions, number of observations per double knockdown and effect size for epistasis. We find that, within ranges that are realistic to real RNAi data, both approaches are capable of inferring pairwise epistasis with favourable precision and sensitivity when only a small number of genes are involved in interactions. In several tests `glinternet` continued to infer correct interactions up to several thousand genes, however the run time prohibits more thorough testing. To demonstrate the model on a real data set, we use the perturbation data from (Rämö et al. 2014). Using `glinternet`, we search for interactions between kinases, and report the most significant results.

Our simulations are performed using `R`, and the source code is available at: `https://github.com/bioDS/xyz-simulation`.

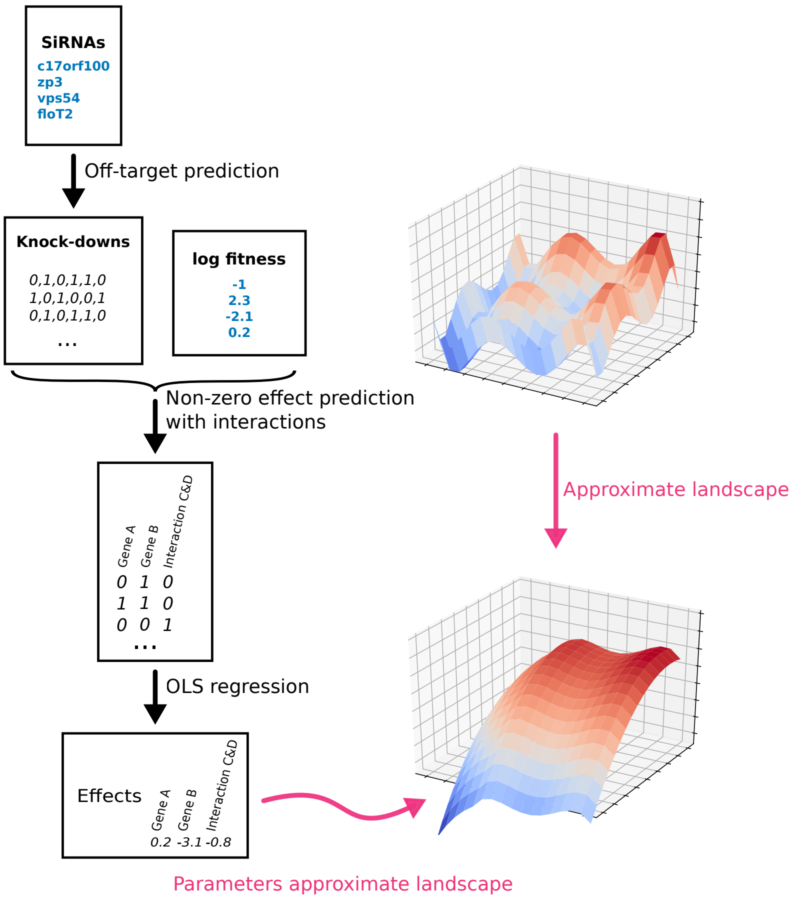

Figure 1. RNAi fitness landscape model. Black arrows indicate outputs that are actually produced. Red arrows indicate theoretical output.

1. Methods

We fix the binary alphabet $\Sigma = \{0, 1\}$ representing the two possible states in a perturbation experiment. The value zero denotes the normal state of the gene (unperturbed wild type), whereas the value one indicates knockdown of the gene (perturbed). For p genes we denote by Σ^p the set of binary sequences of length p, indicating the perturbation status of each gene. Any subset $\mathcal{P} \subseteq \Sigma^p$ is called a perturbation space and its elements are called perturbation types. If the perturbations are genetic mutations, then the perturbation types are genotypes.

1.1. **Fitness landscapes and epistasis.** In the following, we focus on fitness landscapes, but would like to note that the theory also holds for any mapping of perturbation type to phenotype. A fitness landscape is a mapping $f : \mathcal{P} \to \mathbb{R}_+$ from perturbation type space to non-negative fitness values. Genetic interactions are a property of the underlying fitness landscape

(Beerenwinkel, Pachter, and Sturmfels 2007). For $p = 2$ genes, the perturbation type space $\mathcal{P} = \{0, 1\}^2$ contains the wild-type 00, two single perturbations 01 and 10, and the double perturbation 11. The fitness landscape $f : \{0,1\}^2 \to \mathbb{R}_+$ can be written as

$$\begin{aligned} f(0,0) &= \beta_0 \\ f(1,0) &= \beta_0 + \beta_1 \\ f(0,1) &= \beta_0 + \beta_2 \\ f(1,1) &= \beta_0 + \beta_1 + \beta_2 + \beta_{1,2} \end{aligned}$$

for parameters $\beta_i \in \mathbb{R}$. β_0 is called the bias, β_1 and β_2 main effects, and $\beta_{1,2}$ the interaction. Epistasis is defined as

$$\varepsilon = f(0,0) + f(1,1) - f(0,1) - f(1,0) \tag{1}$$

It measures the deviation of the fitness of the double knockdown from the expectation under a linear fitness model in the main effects. We see that $\varepsilon = \beta_{1,2}$.

1.1.1. *Fitness landscape model.* It is challenging to generalize the notion of epistasis (Equation 1), because in higher dimensions, many more types of genetic interactions exist (Beerenwinkel, Pachter, and Sturmfels 2007), even when restricting to pairwise interactions. In general, it will be impossible to estimate all interactions encoded in the fitness landscape reliably from data. In the following, we show how to assess marginal and conditional pairwise epistasis. For $p \geq 1$ genes, we consider the Taylor expansion of the fitness landscape

$$f(x_1, \ldots, x_p) = \beta_0 + \sum_i x_i \beta_i + \sum_{i<j} x_i x_j \beta_{i,j} + \sum_{i<j<k} x_i x_j x_k \beta_{i,j,k} + \ldots \tag{2}$$

Ignoring interactions of order 3 and higher we obtain the more computationally tractable approximation:

$$f(x_1, \ldots, x_p) \approx \beta_0 + \sum_i x_i \beta_i + \sum_{i<j} x_i x_j \beta_{i,j} \tag{3}$$

1.1.2. ~~*Conditional epistasis.* For two genes i and j and a fixed set of background perturbations $b = (x_1, \ldots, x_{i-1}, x_{i+1}, \ldots, x_{j-1}, x_{j+1}, \ldots, x_p) \in \mathbb{R}_+^{p-2}$ we define conditional epistasis between~~

gene i and j given b as

$$\varepsilon_{i,j|b} = f(x_1, \ldots, x_{i-1}, 0, x_{i+1}, \ldots, x_{j-1}, 0, x_{j+1}, \ldots, x_n)$$
$$+ f(x_1, \ldots, x_{i-1}, 1, x_{i+1}, \ldots, x_{j-1}, 1, x_{j+1}, \ldots, x_p)$$
$$- f(x_1, \ldots, x_{i-1}, 1, x_{i+1}, \ldots, x_{j-1}, 0, x_{j+1}, \ldots, x_p)$$
$$- f(x_1, \ldots, x_{i-1}, 0, x_{i+1}, \ldots, x_{j-1}, 1, x_{j+1}, \ldots, x_p)$$

For the fitness landscape model (3), the interaction terms $\beta_{i,j}$ are independent of b and equal to conditional epistasis, that is, $\varepsilon_{i,j|b} = \beta_{i,j}$. Without loss of generality, we can consider $(i, j) = (1, 2)$. Let $b = (x_3, \ldots, x_p)$. In model (3) we have

$$\begin{aligned}\varepsilon_{1,2|b} = {} & f(0, 0, x_3, \ldots, x_p) + f(1, 1, x_3, \ldots, x_p) \\ & - f(1, 0, x_3, \ldots, x_p) - f(0, 1, x_3, \ldots, x_p) \\ = {} & \beta_0 + \left(\beta_0 + \beta_1 + \beta_2 + \beta_{1,2} + \sum_{i>2} x_i \beta_{1,i} + \sum_{i>2} x_i \beta_{2,i}\right) \\ & - \left(\beta_0 + \beta_1 + \sum_{i>2} x_i \beta_{1,i}\right) - \left(\beta_0 + \beta_2 + \sum_{i>2} x_i \beta_{2,i}\right)\end{aligned}$$

All terms except the interaction $\beta_{1,2}$ cancel out, therefore $\varepsilon_{1,2|b} = \beta_{1,2}$.

1.1.2. *Marginal epistasis.* The marginal fitness landscape of genes i and j is

$$f_{i,j}(x_i, x_j) = \sum_{\{x_k \in \{0,1\} | k \neq i,j\}} f(x_1, \ldots, x_p)$$

and marginal epistasis between genes i and j is the epistasis of the marginal fitness landscape,

$$\varepsilon_{i,j} = f_{i,j}(0, 0) + f_{i,j}(1, 1) - f_{i,j}(1, 0) - f_{i,j}(0, 1)$$

For example, for $p = 3$ genes, marginal epistasis between gene 1 and 2 is

$$\begin{aligned}\varepsilon_{1,2} = {} & [f(0, 0, 0) + f(0, 0, 1)] + [f(1, 1, 0) + f(1, 1, 1)] \\ & - [f(1, 0, 0) + f(1, 0, 1)] - [f(0, 1, 0) + f(0, 1, 1)].\end{aligned}$$

For the fitness landscape model (3), the interaction terms $\beta_{i,j}$ are related to marginal epistasis via $\varepsilon_{i,j} = 2^{p-2}\beta_{i,j}$.

~~From Proposition 1 we have that conditional epistasis for a pair of genes (i, j) and a fixed genetic background of the remaining $p-2$ genes equals $\beta_{i,j}$. There are 2^{p-2} such genetic backgrounds, and the conditional epistasis is the same for all of them.~~

~~Thus,~~ We show in appendix A that in the fitness landscape model (3), which contains all main effects and pairwise interactions, but no interactions of higher order, the interaction terms $\beta_{i,j}$ alone determine conditional and marginal epistasis of the fitness landscape.

1.2. **Estimation of epistasis from RNAi perturbation screens.** In *in vitro* RNAi experiments cells are perturbed by reagents, such as siRNA, shRNA, and dsRNA (Singh, Narang, and Mahato 2011), each targeting a specific gene for knockdown. In recent years, it has been shown (Jackson et al. 2006) that siRNAs exhibit strong sequence-dependent off-target effects, such that, in addition to the intended target gene, hundreds of other genes are knocked down. Thus, we can regard siRNA perturbation experiments as combinatorial knockdowns affecting multiple genes simultaneously. On the basis of the fitness landscape model (3), we propose a regression model for the estimation of epistasis from RNAi data. This inference is only feasible because of the unintended combinatorial nature of siRNA knockdowns.

1.2.1. *Perturbation type space.* For an RNAi-based perturbation screen, the perturbation type space $\mathcal{P} = \{g_1, \ldots, g_n\}$ is represented as the $n \times p$ matrix $\boldsymbol{X}$ that contains g_i in row i. Based on the nucleotide sequences of the reagents, perturbations can be predicted by models for micro RNA (miRNA) target prediction (Lewis et al. 2003). We use $\boldsymbol{X}_1, \ldots, \boldsymbol{X}_p$ to denote the p column vectors of $\boldsymbol{X}$ for genes $1, \ldots, p$ and denote by $\boldsymbol{X}_i \circ \boldsymbol{X}_j$ the column vector consisting of the element-wise products of the entries of $\boldsymbol{X}_i$ and $\boldsymbol{X}_j$. As a measure of fitness, we use the vector $\boldsymbol{Y} \in \mathbb{R}^n_+$, denoting the number of cells present after siRNA knockdown.

1.2.2. *Regression model.* We aim to estimate the conditional epistasis $\beta_{i,j}$ between the $\binom{p}{2}$ pairs of genes $(i, j) \in \{1, \ldots, p\}^2$ from all combinatorial gene perturbations in the screen represented in the $n \times p$ matrix $\boldsymbol{X}$, and the $n \times 1$ vector of fitness phenotypes $\boldsymbol{Y}$. Based on (3) we regress phenotype Y on perturbations X,

$$\mathbb{E}\left[\boldsymbol{Y} \mid \boldsymbol{X}\right] = \beta_0 + \sum_i \boldsymbol{X}_i \beta_i + \sum_{i<j} (\boldsymbol{X}_i \circ \boldsymbol{X}_j)\beta_{i,j} \tag{4}$$

The estimated $\beta_{i,j}$ are interpreted as the expected change in the response variable $\boldsymbol{Y}$ per unit change in the predictor variable $(\boldsymbol{X}_i \circ \boldsymbol{X}_j)$ with all other predictors held fixed (Mosteller and Tukey 1977). From Corollary 1 it follows that estimates for marginal epistasis $\varepsilon_{i,j}$ can be obtained by multiplication of $\beta_{i,j}$ with the constant 2^{p-2}.

1.2.3. *Inference.* We aim to infer the regression parameters $\boldsymbol{\beta} = \left(\beta_0, \boldsymbol{\beta}_{\{i:i>0\}}, \boldsymbol{\beta}_{\{i,j:i<j\}}\right)$. Since it is infeasible to directly perform least squares linear regression on the matrix containing all $\binom{p}{2}$ interactions, we use a two-stage process. First, we use either the group lasso regularisation package `glinternet` (Lim and Hastie 2015), or the `xyz` interaction search algorithm (Thanei,

Meinshausen, and Shah 2018) to select non-zero interactions. This variable selection step is the main computational challenge.

When using `glinternet`, we infer parameters $\boldsymbol{\beta} = \left(\beta_0, \boldsymbol{\beta}_{\{i:i>0\}}, \boldsymbol{\beta}_{\{i,j:i<j\}}\right)$ by minimising the squared-error loss function

$$\mathcal{L}(\boldsymbol{Y},\, \boldsymbol{X}; \boldsymbol{\beta}) = \frac{1}{2}\left\|\boldsymbol{Y} - \left(\beta_0 + \sum_i \boldsymbol{X}_i\beta_i + \sum_{i<j}(\boldsymbol{X}_i \circ \boldsymbol{X}_j)\beta_{i,j}\right)\right\|_2^2 \tag{5}$$

under the *strong hierarchy* constraint

$$\beta_{i,j} \neq 0 \Rightarrow \beta_i \neq 0 \text{ and } \beta_j \neq 0. \tag{6}$$

This constraint allows conditional epistasis between gene i and j, i.e., $\beta_{i,j} \neq 0$, only if both single-gene effects β_i and β_j are present and constrains the search space. Lim and Hastie (2015) show that this model can be formulated as a linear regression model with overlapped group lasso (OGL) penalty (Jacob, Obozinski, and Vert 2009), where, in contrast to the group lasso (Yuan and Lin 2006), each predictor can be present in multiple groups.

To perform the variable selection, `xyz` searches for pairs (i, j) that maximise $Y^T X_i X_j$. These are the interaction effects that account for the largest component of the response Y. While `xyz` can be used directly to find the largest interactions, we used `xyz_regression` to estimate all interactions. `xyz_regression` solves the following elastic-net problem (Thanei, Meinshausen, and Shah 2018)

$$\min_{(\beta_0,\beta)\in\mathbb{R}^{p+1},\theta\in\mathbb{R}^{p(p+1)/2}} \left[\frac{1}{2n}\sum_{i=1}^{N}(y_i - \beta_0 - x_i^T\beta - w_i^T\theta)^2 + \lambda(P_\alpha(\beta) + P_\alpha(\theta))\right], \tag{7}$$

where

$$W \in \mathbb{R}^{n\times p(p+1)/2} = (X_1 \circ X_1, X_1 \circ X_2, \ldots, X_1 \circ X_p, X_2 \circ X_2, \ldots, X_p \circ X_p) \tag{8}$$

is the matrix of interactions, and

$$P_\alpha(\beta) = (1-\alpha)\frac{1}{2}||\beta||_{\ell_2}^2 + \alpha||\beta||_{\ell_1} \tag{9}$$

is the elastic-net penalty.

The parameter α decides the compromise between the ridge-regression penalty ($\alpha = 0$) and the lasso penalty ($\alpha = 1$). We left the default value of $\alpha = 0.9$. The solution is found iteratively, with only a particular set of beta values are allowed to be non-zero at each iteration. In every iteration, the beta values that violate the Karush–Kuhn–Tucker conditions are added to this set. Rather than being computed directly, these beta values are found using the `xyz` algorithm (See appendix D for details). We followed the recommendation in (Thanei, Meinshausen, and

Shah 2018) and used $L = \sqrt{p}$ projections to find the strong interactions. Our own tests in Appendix E also suggest that further projections do not improve performance.

Second, once the non-zero effects have been estimated using either `glinternet` or `xyz`, we construct a matrix X' with all elements of the set $\{\boldsymbol{X}_i | \boldsymbol{X}_i \neq \mathbf{0}\} \cup \{\boldsymbol{X}_i \circ \boldsymbol{X}_j | \boldsymbol{X}_i \cdot \boldsymbol{X}_j \neq 0\}$ as columns, in an arbitrary order. We then fit $Y \sim X'\beta$ using R's `lm` least squares linear regression to calculate the coefficient estimates and corresponding p-values, the latter being whether the value significantly deviates from zero according to a t-test with $n-k-1$ degrees of freedom, where k is the number of effects predicted to be non-zero and including in the final regression step. We adjust the p-value to control the false discovery rate with the method of Benjamini and Hochberg (1995), and refer to this adjusted value as the q-value. Given this two-step procedure, we do not expect these values to be the same as if they were calculated using the complete interaction matrix. We are nonetheless able to distinguish between more and less significant effects, with the caveat that the $p < 0.05$ cut-off is completely arbitrary.

1.3. **Software.** The overlapped group lasso for strongly hierarchical interaction terms is implemented in the R-package `glinternet` 1.0.10 by Lim and Hastie (Lim and Hastie 2015) and available through the *Comprehensive R Archive Network* (CRAN) at `https://cran.r-project.org/web/packages/glinternet/`. The `xyz` algorithm is implemented in `xyz` 0.2 by Gian-Andrea Thanei (Thanei, Meinshausen, and Shah 2018) available at `https://cran.r-project.org/web/packages/xyz/`. The simulations are run using a version of this software that also contains a trivial bug fix, available at `https://github.com/bioDS/xyz-simulation`. For the data simulation, analysis and visualisation, we used the R-packages `Matrix` 1.2.6, `dplyr` 0.4.3, `tidyr` 0.4.1 and `ggplot2` 2.1.0. All simulations are performed using `R` 3.2.4.

1.4. **Simulation of RNAi data.** The data simulation followed a three-step procedure. First, we simulate the siRNA–gene perturbation matrix $\boldsymbol{X}$ based on real siRNA libraries. Second, main effects β_i and conditional epistasis between pairs of genes $\beta_{i,j}$ are sampled. Based on $\boldsymbol{X}$ and $\boldsymbol{\beta}$, we then sample fitness phenotypes $\boldsymbol{Y}$ from our model (3) and add noise to match specific signal-to-noise ratios (Hastie, Tibshirani, and Friedman 2009)

$$\text{SNR} = \frac{\text{Var}\left(\mathbb{E}\left[\boldsymbol{Y} \mid \boldsymbol{X}\right]\right)}{\text{Var}\left(\boldsymbol{Y} - \mathbb{E}\left[\boldsymbol{Y} \mid \boldsymbol{X}\right]\right)}. \tag{10}$$

Details for each step including parameter ranges are as follows.

We simulate siRNA–gene perturbation matrices based on four commercially available genome–wide libraries for 20 822 human genes from Qiagen with an overall size of 90 000 siRNAs. First, we predict sequence dependent off-targets using TargetScan (Garcia et al. 2011) for each siRNA as described in (Schmich et al. 2015). We threshold all predictions to be 1 if larger than zero and 0 otherwise. Then, we sample $n = 1\,000$ siRNAs from $\{1, \ldots, 90\,000\}$ and $p = 100$ genes from $\{1, \ldots, 20\,822\}$ without replacement and construct the $n \times p$ binary matrix $\boldsymbol{X}$. Hence, each row $\boldsymbol{X}_{i\cdot}$ then contained the perturbation type $g_i = (x_{i,1}, \ldots, x_{i,p})$.

We simulate $q \in \{5, 20, 50, 100\}$ non-zero conditional epistasis terms $\beta_{i,j}$ between genes i and j from all observed combinatorial knockdowns, i.e. if the simulated screen contained siRNAs that target both genes. This is a necessary condition for the identifiability of $\beta_{i,j}$, as otherwise, according to the model (4), $\beta_{i,j}$ will be multiplied by a zero vector $\boldsymbol{X}_i \circ \boldsymbol{X}_j = \mathbf{0}$. The effect size of the $\beta_{i,j}$ is sampled from Norm$(0, 2)$. In order to maintain a strong hierarchy, we subsequently simulate for each interaction $\beta_{i,j}$ both main effects β_i and β_j. Further, we add $r \in \{0, 20, 50, 100\}$ additional main effects. The effect sizes of the main effects are sampled from Norm$(0, 1)$, so that the variance in the response fitness phenotypes are split in a ratio of 1:2 between main effects and interactions.

In order to model synthetic lethal pairs, interactions with effect strength of -1000 (on log scale) are added to the simulated data. Since lethal interactions may occur with little or no main effect present (Jerby-Arnon et al. 2014), we allow these pairs to violate the strong hierarchy and do not add main effects. This is done both for biological plausibility, and to evaluate the performance of `xyz` and `glinternet` under less ideal circumstances. Since only `glinternet` assumes the strong hierarchy, this scenario might favour `xyz`.

Based on simulated perturbation matrices $\boldsymbol{X}$, simulated main effects β_i and interaction terms $\beta_{i,j}$, we sampled fitness values with $\beta_0 = 0$ according to the fitness landscape model (3)

$$\boldsymbol{Y} \sim \text{Norm}\left(\textstyle\sum_i \boldsymbol{X}_i\beta_i + \sum_{i<j}(\boldsymbol{X}_i \circ \boldsymbol{X}_j)\beta_{i,j},\, \sigma^2\boldsymbol{I}\right),$$

where we chose σ^2 for fixed SNRs $s \in \{2, 5, 10\}$.

1.5. **Evaluation criteria.** We focus the evaluation on the estimated parameters of the model, specifically the conditional epistasis terms, $\hat{\boldsymbol{\beta}}_{\{i,j:i<j\}}$, rather than the model's performance in predicting the fitness phenotypes $\boldsymbol{Y}$. Given the ground truth of true conditional epistasis between gene i and j, $\boldsymbol{\beta}_{\{i,j:i<j\}}$, we assess the performance of the model to identify epistasis, i.e., estimated non-zero coefficients $\hat{\beta}_{i,j}$, by computing the number of true positives (TPs), false positives (FPs) and false negatives (FN). Here, TPs represent the number of gene pairs (i, j) such that $\beta_{i,j} \neq 0$ and $\hat{\beta}_{i,j} \neq 0$, FPs the number of gene pairs $(i, j) : \beta_{i,j} = 0$ and $\hat{\beta}_{i,j} \neq 0$ and FNs the number of gene pairs $(i, j) : \beta_{i,j} \neq 0$ and $\hat{\beta}_{i,j} = 0$. The performance is then summarised using the following measures

$$\begin{aligned}
\text{precision} &= \frac{\text{TP}}{\text{TP} + \text{FP}} \\
\text{recall} &= \frac{\text{TP}}{\text{TP} + \text{FN}} \\
\text{F1} &= 2\frac{\text{precision} \times \text{recall}}{\text{precision} + \text{recall}}
\end{aligned}$$

Furthermore, we investigate whether estimates $\hat{\beta}_{i,j}$ have the same sign as the ground truth conditional epistasis and we quantify the deviation of the magnitude from the truth. Where

applicable, we also evaluate the effect of selection of only those $\beta_{i,j}$ which significantly deviate from zero on the model's performance.

2. Results

First, we evaluate the proposed approach to estimating epistatic effects from off-target perturbations on simulated data. The approach depends on a model able to detect non-zero pairwise interactions (Figure 1). Here, we evaluate the approach using two such alternative models, `glinternet` and `xyz`.

We evaluate the ability of both `xyz` and `glinternet` to identify epistasis between pairs of genes from RNAi screens on simulated data with $p = 100$ genes and $n = 1\,000$ siRNAs. Only for `xyz`, we also test larger data sets, with $p = 1\,000$ and $n = 10\,000$. We use off-target information from real siRNAs and investigate the performance for varying signal-to-noise ratios, number of true interactions, number of observations per double knockdown, and effect sizes for epistasis.

We perform a separate set of tests where we specifically assess the performance of the two methods to identify synthetic lethal interactions, the strongest negative interactions. For this purpose, we simulate a separate data set that contains additional synthetic lethal pairs of genes. In this test, we attempt to identify only lethal interactions using `xyz` and `glinternet`, given increasingly large numbers of genes.

2.1. Identification of epistasis under varying conditions.

Both `xyz` and `glinternet` are tested on a series of small simulated data sets. For each combination of parameters $q \in \{5, 20, 50, 100\}$, $r \in \{0, 20, 50, 100\}$ and $s \in \{2, 5, 10\}$, controlling the number of true interactions, the number of additional main effects, and the SNRs of the fitness phenotypes, respectively, we sample 50 independent data sets. `xyz` is tested on a series of larger data sets, with parameters $q \in \{50, 200, 500, 1000\}$, $r \in \{0, 200, 500, 1000\}$ and $s \in \{2, 5, 10\}$. Only 10 independent data sets are sampled in these cases. Each data set consists of the perturbation matrix $\boldsymbol{X}$, phenotypes $\boldsymbol{Y}$, true conditional epistasis $\beta_{i,j}$ and main effects β_i.

The distribution of the number of observations for pairwise knockdowns of gene i and j is shown in Appendix, Figure 10 for an exemplary perturbation matrix $\boldsymbol{X}$. While only a few genes have many observations, 87% of gene pairs are simultaneously perturbed by at least one siRNA. Note that the distribution seen in Figure 10 is similar for both $p = 100$ and $p = 1,000$ genes. We also find that number of additional main effects has relatively little impact on detecting interactions (Appendix B), and this value is kept constant during our tests. We select only estimates $\hat{\beta}_{i,j}$ with a magnitude significantly different from zero (q-value < 0.05). This significantly improves precision, at a slight cost to recall, using both `glinternet` and `xyz` (Appendix, Figure 13).

2.1.1. *Number of double knockdowns per gene pair.*

We fixed the number of additional main effects to 20 and investigated performance with respect to the number of double knockdowns per epistatic gene pair, i.e. siRNAs that target both genes (Figure 2). The results are largely

similar for both `xyz` and `glinternet`. As expected, for increasing numbers of observations, we observe an increase in precision and recall with a steeper increase of precision compared to recall and decreased performance for higher number of true interactions. The number of true epistatic gene pairs primarily affects recall, which decreases for higher numbers of true non-zero $\beta_{i,j}$. For gene pairs with more than 80 observations of the double knockdown, `glinternet` shows strong performance with F1 values between $0.68 - 0.9$ across all tested numbers of true interactions and an SNR larger than or equal to 5 (Figure 2a).

`xyz` shows significantly improved performance for gene pairs with more than 40 observations, with F1 values almost all above 0.25. Small numbers of true interactions are particularly accurate, with $F1 > 0.5$ when there are also only 5 such effects (Figure 2b).

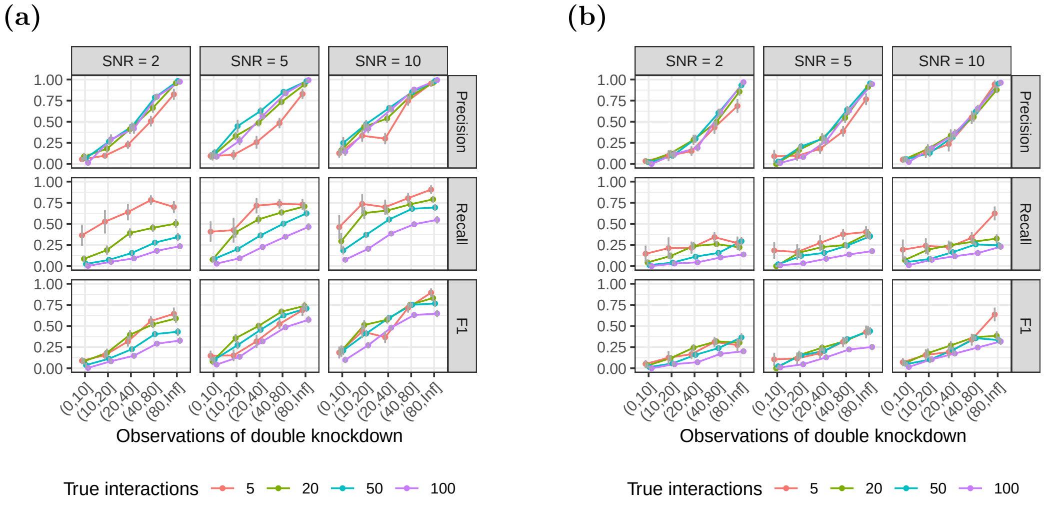

Figure 2. Identification of epistasis for increasing numbers of observations of the pairwise double knockdown. The number of additional main effects not overlapping with the set of interacting genes is fixed to 20. Results using (a) `glinternet` and (b) `xyz`.

The number of times each pair of genes is observed is shown in Figure 3. We see that in the large simulation, in which all parameters are multiplied by ten, the number of observations of each pair of genes is similarly scaled. As a result, the overall distribution is similar to the smaller simulation.

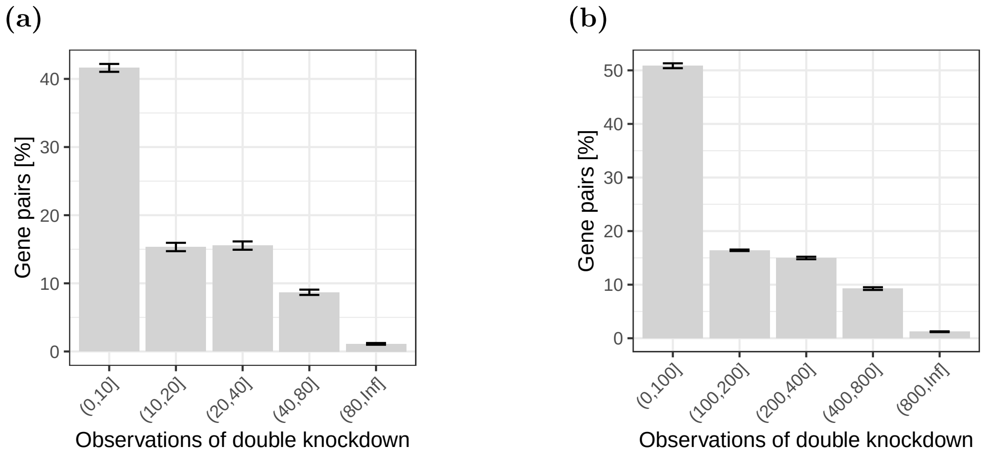

Figure 3. The distribution of the fraction of gene pairs stratified by ranges of observed double knockdowns. Gene pairs with zero observations are not shown. (a) $p = 100,\ n = 1000$; (b) $p = 1000,\ n = 10000$.

2.1.2. *Epistatic effect size.* We observe that, for both `xyz` and `glinternet`, the performance of the model increases with the absolute value of the magnitude of the conditional epistasis between pairs of genes $|\beta_{i,j}|$ (Figure 4). Both for negative and positive epistasis, recall and precision steeply increase with increasing effect size. For pairs of genes with $|\beta_{i,j}| > 1$ and SNRs ≥ 10, the model performs favourably with F1 values of 0.6 and higher in `glinternet`, and at least 0.25 in `xyz`. Overall performance also marginally improves for `glinternet` at SNR $= 5$, but no clear effect is seen for `xyz` or SNR $= 10$. With both `xyz` and `glinternet`, we observe exceptions to the general pattern of the overall V-shape for precision and recall, where strongly negative and positive epistasis and weak epistasis lead to high and low performance of the model, respectively. This effect can be explained by the fact that, after the significance test, an extremely small number of interactions are reported in these ranges (most often only one), with no false positives. The fact that the model's performance notably decreases for small effect sizes around zero explains why we observe a trend of decreasing performance for increasing numbers of true interactions, when we average over all effect sizes. This is because sampling true epistatic effect sizes from $\mathrm{Norm}(0, 2)$ for increasing numbers of true interactions increases the fraction of interactions with small effects around zero.

Notably, we can see in Figure 4b that even when the overall performance is poor, `xyz` is still able to find a small number of strong interactions relatively accurately. This is particularly promising, since synthetic lethal pairs would be such interactions.

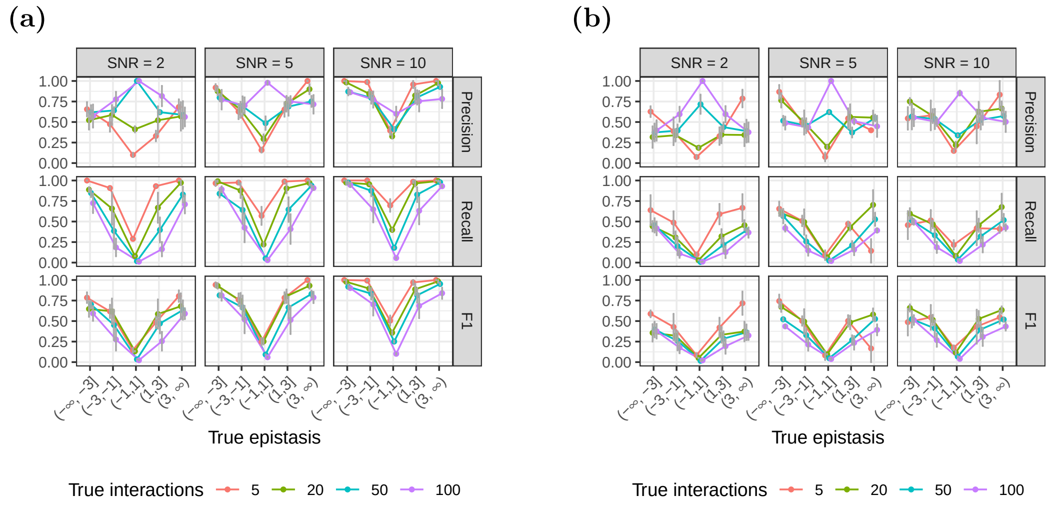

Figure 4. Identification of epistasis for varying effect size. Using (a) `glinternet` and (b) `xyz`.

2.1.3. *Direction.* We evaluate the ability of each method to distinguish between negative and positive epistasis among epistatic gene pairs identified as true positives (Figure 5). For both `glinternet` and `xyz`, the fraction of incorrect estimates of direction (positive vs. negative) is higher for decreasing effect size and increasing number of true interactions. For epistatic effects with an absolute value > 1, we observe at most 3% incorrect predictions with `glinternet`, and 8% with `xyz`. We observe at most 9% and 15% incorrect predictions for smaller effect sizes for `glinternet` and `xyz` respectively. Furthermore, we observe that increasing SNRs leads to a subtle decrease of incorrectly predicted direction.

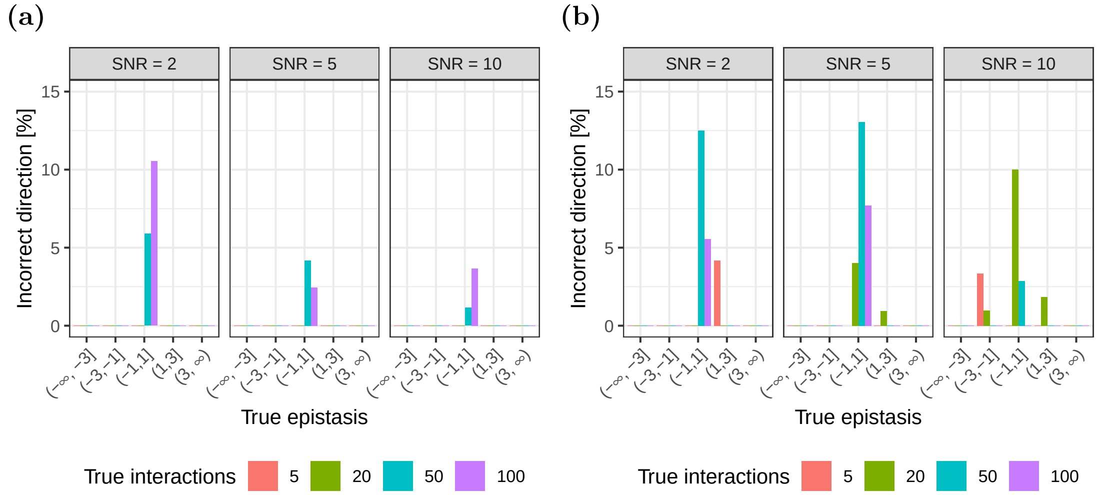

Figure 5. Concordance between the sign of true and estimated epistasis. The fraction of incorrectly identified signs between true and estimated epistasis for (a) `glinternet` and (b) `xyz`.

2.1.4. *Magnitude.* We evaluate the deviation of the magnitude of estimates for epistasis from the ground truth as a function of observed double knockdowns (Appendix, Figures 14 and 21). The deviation in magnitude is computed as $\frac{|\beta_{i,j}|-|\hat{\beta}_{i,j}|}{|\beta_{i,j}|}$, i.e. the percent relative change in deviation with respect to the true epistasis. We observe that across varying numbers of observations the model predicts the magnitude of epistasis between pairs of genes with high accuracy using both `xyz` and `glinternet`.

2.2. **Scalability.** Running `glinternet` until it has converged takes a prohibitively long time on larger data sets. While we are able to run our $p = 100, n = 1,000$ simulations in slightly under two minutes, increasing to $p = 1,000, n = 10,000$ takes over two days using ten cores. Although using more threads is possible, the running time is already dominated by single-threaded components with ten cores. The multi-threaded performance is therefore limited to by Ahmdahl's Law to approximately the performance we see here. Since fitting with small lambda values takes the majority of the time, we can improve this by changing the minimum value of lambda that gets used. Adjusting this from $\frac{\texttt{lambdaMax}}{100}$ to $\frac{\texttt{lambdaMax}}{4}$, and fitting only five lambdas in this range rather than fifty, `glinternet` still takes over an hour. This makes the repeated simulations from subsection 2.1 impractical at a larger scale with `glinternet`, although we do investigate some larger data sets in subsection 2.3.

Since `xyz` has significantly shorter run time than `glinternet`, here we more thoroughly investigate performance on larger data sets. Repeating the earlier simulations with every parameter increased by a factor of 10 (Figure 6), we find that the overall trends remain the same. The fraction of incorrectly identified signs is omitted, as in this test there are no such results.

There is a significant drop in both precision and recall, and now only effects with a magnitude greater than 3 are found a significant amount of the time (Figure 6b).

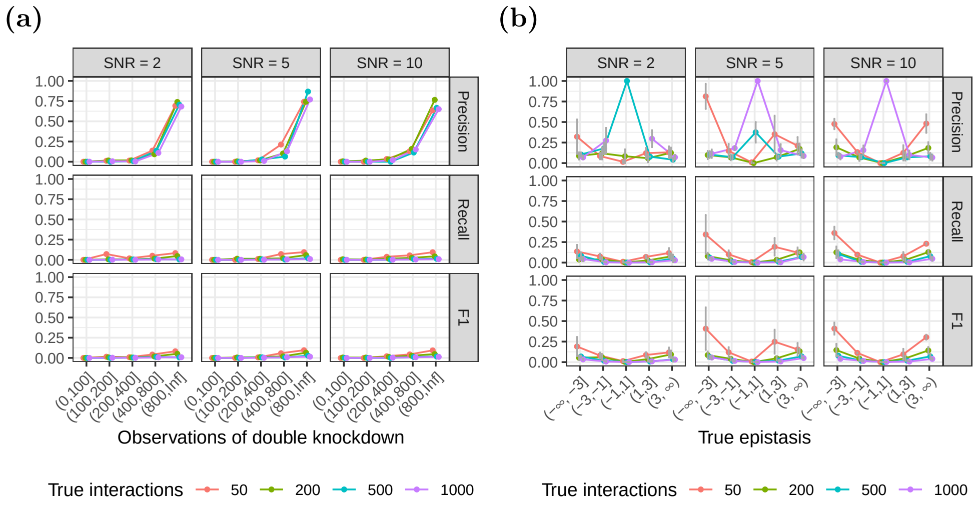

Figure 6. Simulations repeated using `xyz` and larger data sets. (a) number of observations of double knockdown. (b) Precision/recall/f1 by actual effect strength.

2.3. **Synthetic Lethal Pairs.** Synthetic lethal pairs are of particular interest, and given that `xyz` is able to somewhat reliably find extremely strong interactions, it is natural to ask whether it can be used to quickly find lethal pairs, despite its poor performance on weaker interactions. We fix the number of main effects to 10, and simulated 10 000 siRNAs on 1 000 genes. Synthetic lethal pairs are created as interaction effects of magnitude −1000 (log scale). This rather extreme assumption makes synthetic lethals the best possible case for detection via regression. In practice, synthetic lethal detection accuracy would likely be somewhere between what we see here and that of a small negative effect. Since lethal pairs often do not have strong main effects (i.e. do not follow the strong hierarchy assumption), the components of the interaction are *not* used as main effects in this case.

Increasing the number of lethal interactions significantly reduces recall, but does not have a clear effect on precision. At this scale, `xyz` is often able to correctly identify some lethal interactions (Figure 7), particularly when there are only a few to find.

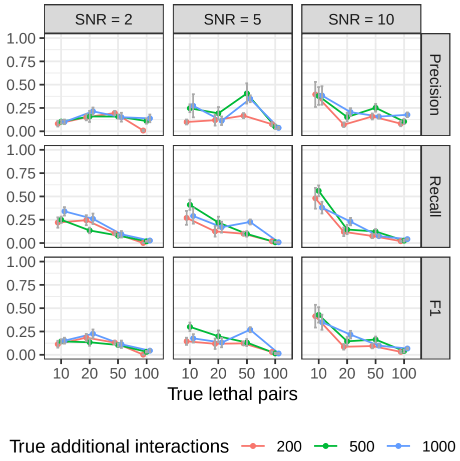

Figure 7. Precision, recall, and F1 performance for varying numbers of synthetic lethal pairs, with additional background interactions, using `xyz`. Neither side of the lethal interactions are used as main effects, and as far as lethal interactions are concerned, there is no hierarchy present.

2.3.1. *Synthetic lethality detection in larger matrices.* While we could not run a significant number of tests at this scale using `glinternet`, we could investigate how well its accuracy scales compared to `xyz`. To do this, we simulated sets of up to $p = 4000$ genes, and measured the performance of both `xyz` and `glinternet`. In this case, both to avoid allocating more elements to a matrix than R allows, and to keep the run time of `glinternet` low, only $n = 2 \times p$ siRNAs are simulated. The ratio of siRNAs, genes, main effects, interactions, and lethals, is fixed to: $n = 200$ siRNAs, $p = 100$ genes, $b_i = 1$ main effect, $b_{ij} = 20$ interaction effects, $l = 5$ lethal interactions. Data sets are then generated with these values multiplied by 5, 10, 20, and 40. As in the previous simulation, components of lethal interactions are *not* added as main effects. The strong hierarchy assumption is not valid in this case.

Interactions are then found with both `xyz` and `glinternet`. Here we focus specifically on synthetic lethal detection, and only correct lethal pairs are considered true positives, Any other pair (including a true interaction that is not a lethal) is considered a false positive.

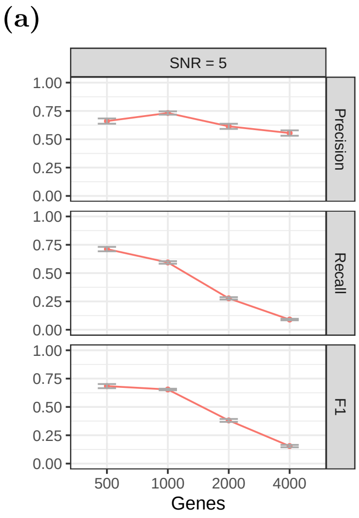

Figure 8. The performance of (a) `glinternet` and (b) `xyz` on increasingly large data sets.

We can see in Figure 8a that precision with `glinternet` remains fairly consistent as p increases. There is a roughly proportional reduction in recall as the number of lethal interactions increases. After a slight increase from 500 to 100 genes, the actual number of significant interactions found remains fairly consistent. Beyond $p = 2000$, we found that `xyz` typically fails to find any of the lethal pairs (Figure 8b)

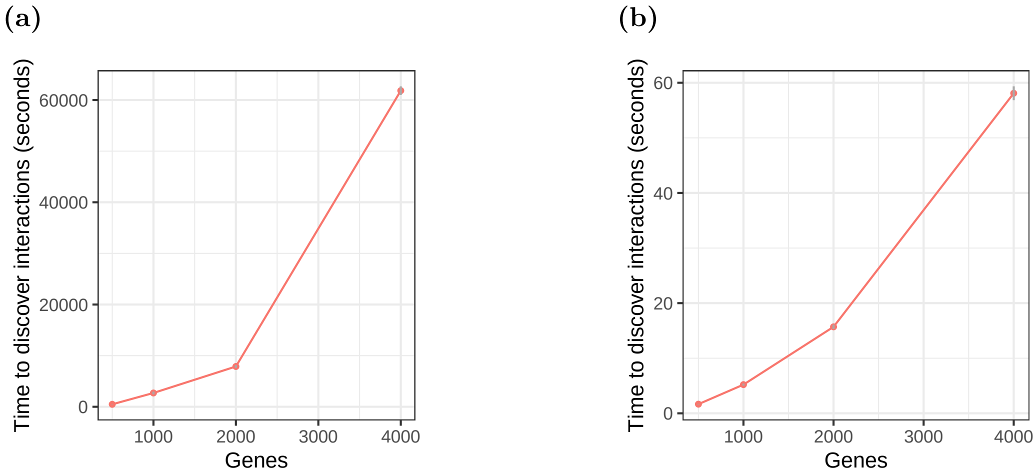

Figure 9. Run time in seconds to find interactions on increasingly large data set. 9a: `glinternet`. 9b: `xyz`. We compiled `glinternet` with OpenMP and ran with `numCores = 10`.

Figure 9 shows that neither `xyz` nor `glinternet` quite demonstrate a linear run time, but the run time for `glinternet` increases sharply beyond $p = 2000$. It is possible that this is simply the result of less efficient cache use with larger data, but it is nonetheless worth noting.

2.4. **Violations to model assumptions.** For the regularised regression model (4) we assume strong hierarchy (6) between main effects β_i and interaction terms $\beta_{i,j}$ in order to reduce the search space of all possible non-zero coefficients $p + \binom{p}{2}$ during inference. We refer the reader to (Lim and Hastie 2015), where Lim and Hastie show how violations to this assumption affect the performance. For instance, the performance of the model is evaluated when the ground truth only obeys weak hierarchy, i.e. only one main effect present, no hierarchy, or anti-hierarchy. Additionally, approximately 2.5% of simulations failed to run using `xyz`, because the estimated interaction frequency of non interacting pairs was too low. These were fairly evenly distributed across all combinations of parameters (Figure 16), and are not believed to have substantially affected the results.

2.5. **Summary recommendation.** After simulating siRNA knockdown data sets of various sizes, and under various conditions, and attempting to reconstruct the interacting pairs using both `xyz` and `glinternet`, we arrive at the following recommendations. For data sets containing less than 4,000 genes (assuming between 2 and 10 experiments per gene), we recommend using `glinternet` to find interactions. Where `glinternet` would have a prohibitively long run time (data sets larger than those mentioned above), `xyz` continues to run quickly, and may still identify some useful results (Figure 7), particularly when interactions are observed a large number of times in the data and have strong effects (sections 2.1.1 and 2.1.2). Particularly when one expects a small number of significant interactions, increasing the number of projections beyond $\sqrt{n}$ may improve performance here (see Appendix, Figure 15c).

2.6. **Effects in real data.** Following the recommendation we have arrived at in Section 2.5, we apply `glinternet` (followed by a linear regression analysis) to estimate epistatic effects from a real data set. We use the perturbation data from (Rämö et al. 2014), containing siRNA screens targeting kinases in the presence of five bacterial pathogens and two viruses, and apply the routine as described in Section 1.2 to identify pairwise kinase-kinase interactions. Specifically, we restrict the data to siRNAs that target kinases from the Qiagen Human Kinase siRNA Set V4.1, and the off-target effects within this set, resulting in an input matrix containing 11 214 perturbations × 667 genes. Using $f = log_2(\frac{\text{Cells after}}{\text{Cells before}})$ as a fitness measure, we found 1662 effects, 116 of which had a p-value less than 0.05. Since we have assumed that perturbations are binary in our simulations, we continue to do so here. As a result, all non-zero predicted off-target effects are given a value of 1. The ten most significant predicted effects are shown in Table 1. * Interestingly, the most significantly associated pair of genes, CDK5R1 and RPS6KA2, are both related to a common pathway. Specifically, CDK5R1 activates CDK5,

*The full set of results, significant or otherwise, can be found at `https://github.com/bioDS/xyz-simulation/blob/master/real_data_results_sorted.csv`

which, along with RPS6KA2, is part of the IL-6 signalling pathway (Kandasamy et al. 2010). Searching both the ConsensusPathDB database (Kamburov et al. 2009), and STRING database (Szklarczyk et al. 2017) for relations between the found pairs, we find that a number of the interactions suggested here could be the result of existing known interactions. We each of the identified pairs of genes, we searched for common neighbours (a third gene with which both interact), shared pathways, and whether the produced proteins are found in the same protein complexes, and found the following known relationships:

CDK5R1 and RPS6KA2 share a common neighbour, and are present in four of the same enriched pathway-based sets. TTK and RPS6KA2 share nine common neighbours. RIPK4 and GRK3 share one neighbour,and homologs were found interacting in other species. TNIK and PANK4 share one neighbour, as do MAPK4 and TRPM7, MAP2K6 and UCK1, and HIPK1 and NUAK2. As we could not locate the other identified pairs in the database, we hypothesise that they might constitute novel interactions.

Of the interactions present in Table 1, we see that HIPK1 and NUAK2, TNIK and PANK4, and MAP2K6 and UCK1 are predicted to have negative epistatic effects, and may be promising synthetic lethal candidates.

Gene i	Gene j	Type	~~Estimated~~ Combined Effect	P-value	i effect	j effect
CDK5R1	RPS6KA2	interaction	12.52	0.0047	1.71	-2.32
RIPK4	GRK3	interaction	-3.24	0.0056	-24.5	1.87
PHKB	GUK1	interaction	-7.47	0.0061	6.23	-28.4
MAP2K6	UCK1	interaction	-40.89	0.0094	13.8	-22.6
TNIK	PANK4	interaction	-37.41	0.0115	21.3	5.21
RPS6KB2	TTK	interaction	172.04	0.0118	0.5	-20.4
MAPK4	TRPM7	interaction	9.49	0.0120	8.46	16.3
HIPK1	NUAK2	interaction	-13.17	0.0126	18.1	29.5
CDK19	NA	main	3.80	0.0136	3.80	
C17orf75	MAPK8IP3	interaction	21.74	0.0136	5.91	20.4

Table 1. Ten most significant predicted effects of siRNA perturbation screens, targeting all human kinases.

For comparison we also fit a linear model including all genes, but no interactions. Comparing the R^2 values for each, we find that individual gene effects explain $\approx$ 15% of the variance ($R^2 = 0.150$) Including the interactions chosen by `glinternet`, and removing the main effects it sets to zero, we have $R^2 = 0.392$, more than doubling the fraction of explained variance. This highlights the importance of accounting for interactions in large-scale genotype-phenotype analyses, and relevance of bioinformatic tools with this capability.

3. Discussion

To the best of our knowledge, the presented model is the first approach that leverages the combinatorial nature of RNAi knockdown data resulting from sequence-dependent off-target effects for the large-scale prediction of epistasis between pairs of genes. To do this, we take the second-order approximation of the fitness landscape, including only individual and pairwise effects, and attempt to infer the parameters of this model. Since `glinternet` is able to find pairwise interactions among $p = 1,000$ genes, we speculate that searching for three-way interactions is feasible among $\sqrt[3]{1,000^2} = 100$ genes. We are not aware of any software currently able to do this, however.

For the majority of our tests, we simulate the presence of a strong hierarchy. This constraint would imply that for the inference of non-zero epistatic effects between gene i and j, $\beta_{i,j}$, we penalise cases where the main effects for both single genes, β_i and β_j, are zero. This constraint significantly decreases the complexity of the search space of interactions. However, in biology there are many examples of epistasis where the marginal effects of individual genes are very small, for instance if both genes redundantly execute the same function within the cell (Puchta et al. 2016). Costanzo et al. (2010) found in their study of experimental double knockouts in yeast that single mutants with decreasing fitness phenotypes tended to exhibit an increasing number of genetic interactions. This observation is reassuring for `glinternet`, which can pick up the interaction as long as the true single-mutant effects are not exactly zero. Moreover, Lim and Hastie showed in a simulation study that the model is in fact flexible enough to also identify pairwise interactions violating the strong hierarchy constraint (Lim and Hastie 2015). For the detection of strong interactions, specifically synthetic lethal pairs, we have also demonstrated that the strong hierarchy constraint is not required.

In a simulation study, we sampled perturbations for $n = 1000$ siRNAs and $p = 100$ genes, and $n = 10000$ siRNAs with $p = 1000$ genes. As a consequence of high-throughput genome-wide screening platforms, the setting of $n = 10 \times p$, i.e. ten perturbations with different siRNAs per gene, is realistic even for higher order organisms with tens of thousands of genes (Rämö et al. 2014; Schmich et al. 2015). Sampling the perturbations directly from commercially available RNAi libraries allows us to translate results from the simulation study to applications on real data. We observe that increasing SNRs, as expected, results in an overall increase of the number of successfully identified gene pairs with true epistasis.

Nevertheless, we found that even for a moderate SNR of only 2, the model identifies interactions with acceptable performance using `glinternet` (F1 $>$ 0.5 for 50 true interactions), when we observed each double knockdown over 40 times (Figure 2a) or the effect size of epistasis is larger than 1, i.e. $|\beta_{i,j}| > 1$ (Figure 4a). For an SNR of 5 and across all tested numbers of additional gene pairs and epistatic effect sizes, the performance of the model is approximately constant at around F1 = 0.5, independent of the number of true epistatic gene pairs (Figure 11b).

Performance in our simulations also suggests that `xyz` is unable to accurately identify interactions in large data sets. Although `xyz` has a consistently short run time, and appears capable of running on genome-scale data, we see a significant drop in all other performance measures beyond $p = 1000$ genes.

The results when using `glinternet`, however, suggest that the general approach is able to accurately identify pairwise epistasis from large-scale RNAi data sets, given that the SNR of measured fitness phenotypes is larger than 2 and the effect size of epistasis is larger than 1. It is challenging to compare the performance of these models to approaches that estimate genetic interactions from other data, such as for instance from double knockout experiments (Costanzo et al. 2010), due to different scales of the epistatic effect size, however, the high precision of `glinternet` seems quite competitive. Moreover, our simulations demonstrated that if true epistatic effects between pairs of genes are identified, the model identifies both the direction of epistasis (positive and negative) as well as the magnitude of the epistatic effect with high accuracy (Figures 5 and 14).

In detecting lethal interactions specifically, the high precision of `glinternet` after testing for significant deviations is particularly promising. Using this as a method to detect likely synthetic lethal interactions from RNAi data sets, we could propose candidates for further investigation as anticancer drug targets (Chan and Giaccia 2011)(Ashworth, Lord, and Reis-Filho 2011). While the run time may prevent `glinternet` from being used as such a method in genome-scale applications, we can recommend it for use with smaller data sets, or where the number of potential interactions can be significantly reduced prior to running `glinternet`. As the precision does not appear to suffer with larger input, only the run time, we believe combining linear regression with a perturbation matrix is a promising method for further investigation, and work to improve the performance sufficiently for use in genome-scale applications is ongoing.

Finally, it is worth noting that this approach is not limited to siRNA perturbation matrices, or to synthetic lethal detection. Any method of suppressing gene expression, combined with an affected proxy for fitness, could be used to find likely candidates for epistasis.

Acknowledgements

This work has partially been funded by SystemsX.ch, the Swiss Initiative in Systems Biology, under IPhD grant 2009/025 and RTD grants 51RT-0_126008 (InfectX) and 51RTP0_151029 (TargetInfectX), evaluated by the Swiss National Science Foundation.

We acknowledge support from the Royal Society Te Apārangi through a Rutherford Discovery Fellowship (RDF-UOO1702) awarded to AG. This work was partially supported by Ministry of Business, Innovation, and Employment of New Zealand through an Endeavour Smart Ideas grant (UOOX1912) and a Data Science Programmes grant (UOAX1932).

References

Ashworth, A, CJ Lord, and JS Reis-Filho (Apr. 2011). "Genetic Interactions in Cancer Progression and Treatment". en. *Cell* 145.1, pp. 30–38. ISSN: 0092-8674. DOI: 10.1016/j.cell.2011.03.020.

Beerenwinkel, N, L Pachter, and B Sturmfels (2007). "Epistasis and Shapes of Fitness Landscapes". *Statistica Sinica.*

Benjamini, Y and Y Hochberg (1995). "Controlling the False Discovery Rate: A Practical and Powerful Approach to Multiple Testing". en. *Journal of the Royal Statistical Society: Series B (Methodological)* 57.1, pp. 289–300. ISSN: 2517-6161. DOI: 10.1111/j.2517-6161.1995.tb02031.x.

Chan, DA and AJ Giaccia (May 2011). "Harnessing Synthetic Lethal Interactions in Anticancer Drug Discovery". en. *Nature Reviews Drug Discovery* 10.5, pp. 351–364. ISSN: 1474-1784. DOI: 10.1038/nrd3374.

Cordell, HJ (Oct. 2002). "Epistasis: What It Means, What It Doesn't Mean, and Statistical Methods to Detect It in Humans". en. *Human Molecular Genetics* 11.20, pp. 2463–2468. ISSN: 14602083. DOI: 10.1093/hmg/11.20.2463.

Costanzo, M et al. (2010). "The Genetic Landscape of a Cell." *Science.*

Crawford, L, P Zeng, S Mukherjee, and X Zhou (July 2017). "Detecting Epistasis with the Marginal Epistasis Test in Genetic Mapping Studies of Quantitative Traits". en. *PLOS Genetics* 13.7, e1006869. ISSN: 1553-7404. DOI: 10.1371/journal.pgen.1006869.

de Visser, JAGM, TF Cooper, and SF Elena (Dec. 2011). "The Causes of Epistasis". *Proceedings of the Royal Society B: Biological Sciences* 278.1725, pp. 3617–3624. DOI: 10.1098/rspb.2011.1537.

DepMap, B (June 2020). *DepMap 20Q2 Public.* DOI: 10.6084/m9.figshare.12280541.v4.

Force, T and KL Kolaja (2011). "Cardiotoxicity of Kinase Inhibitors: The Prediction and Translation of Preclinical Models to Clinical Outcomes." *Nature Reviews Drug Discovery.*

Garcia, DM et al. (2011). "Weak Seed-Pairing Stability and High Target-Site Abundance Decrease the Proficiency of Lsy-6 and Other microRNAs". *Nature Structural & Molecular Biology.*

Hanahan, D and RA Weinberg (2011). "Hallmarks of Cancer: The next Generation." *Cell.*

Hastie, T, R Tibshirani, and J Friedman (2009). *The Elements of Statistical Learning.* Springer New York.

Holohan, C, S Van Schaeybroeck, DB Longley, and PG Johnston (2013). "Cancer Drug Resistance: An Evolving Paradigm." *Nature Reviews. Cancer.*

Jackson, AL et al. (Jan. 2006). "Widespread siRNA "off-Target" Transcript Silencing Mediated by Seed Region Sequence Complementarity". en. *RNA* 12.7, pp. 1179–1187. ISSN: 1355-8382, 1469-9001. DOI: 10.1261/rna.25706.

Jacob, L, G Obozinski, and JP Vert (2009). "Group Lasso with Overlap and Graph Lasso". *ICML '09*.

Jerby-Arnon, L et al. (Aug. 2014). "Predicting Cancer-Specific Vulnerability via Data-Driven Detection of Synthetic Lethality". en. *Cell* 158.5, pp. 1199–1209. ISSN: 00928674. DOI: 10.1016/j.cell.2014.07.027.

Kaelin, WG (Sept. 2005). "The Concept of Synthetic Lethality in the Context of Anticancer Therapy". en. *Nature Reviews Cancer* 5.9, pp. 689–698. ISSN: 1474-175X, 1474-1768. DOI: 10.1038/nrc1691.

Kamburov, A, C Wierling, H Lehrach, and R Herwig (Jan. 2009). "ConsensusPathDB-a Database for Integrating Human Functional Interaction Networks". *Nucleic Acids Research* 37, pp. D623–D628. ISSN: 0305-1048. DOI: 10.1093/nar/gkn698.

Kandasamy, K et al. (Jan. 2010). "NetPath: A Public Resource of Curated Signal Transduction Pathways". eng. *Genome Biology* 11.1, R3. ISSN: 1474-760X. DOI: 10.1186/gb-2010-11-1-r3.

Laufer, C, B Fischer, M Billmann, W Huber, and M Boutros (2013). "Mapping Genetic Interactions in Human Cancer Cells with RNAi and Multiparametric Phenotyping." *Nat Meth.*

Lewis, BP, Ih Shih, MW Jones-Rhoades, DP Bartel, and CB Burge (Dec. 2003). "Prediction of Mammalian MicroRNA Targets". en. *Cell* 115.7, pp. 787–798. ISSN: 0092-8674. DOI: 10.1016/S0092-8674(03)01018-3.

Lim, M and T Hastie (2015). "Learning Interactions via Hierarchical Group-Lasso Regularization". en. *J. Comput. Graph. Stat.* 24.3, pp. 627–654.

McDonald, ER et al. (July 2017). "Project DRIVE: A Compendium of Cancer Dependencies and Synthetic Lethal Relationships Uncovered by Large-Scale, Deep RNAi Screening". en. *Cell* 170.3, 577–592.e10. ISSN: 00928674. DOI: 10.1016/j.cell.2017.07.005.

Mosteller, F and JW Tukey (1977). "Data Analysis and Regression: A Second Course in Statistics." *Addison-Wesley Series in Behavioral Science: Quantitative Methods.*

O'Neil, NJ, ML Bailey, and P Hieter (Oct. 2017). "Synthetic Lethality and Cancer". en. *Nature Reviews Genetics* 18.10, pp. 613–623. ISSN: 1471-0064. DOI: 10.1038/nrg.2017.47.

Orr, HA (Aug. 2009). "Fitness and Its Role in Evolutionary Genetics". English. *Nature Reviews. Genetics* 10.8, pp. 531–539.

Otwinowski, J and I Nemenman (May 2013). "Genotype to Phenotype Mapping and the Fitness Landscape of the E. Coli Lac Promoter". en. *PLoS ONE* 8.5. Ed. by AJ Yates, e61570. ISSN: 1932-6203. DOI: 10.1371/journal.pone.0061570.

Poelwijk, FJ, M Socolich, and R Ranganathan (Sept. 2019). "Learning the Pattern of Epistasis Linking Genotype and Phenotype in a Protein". en. *Nature Communications* 10.1, p. 4213. ISSN: 2041-1723. DOI: 10.1038/s41467-019-12130-8.

Puchta, O et al. (May 2016). "Network of Epistatic Interactions within a Yeast snoRNA". en. *Science* 352.6287, pp. 840–844. ISSN: 0036-8075, 1095-9203. DOI: 10.1126/science.aaf0965.

Rämö, P et al. (Dec. 2014). “Simultaneous Analysis of Large-Scale RNAi Screens for Pathogen Entry”. *BMC Genomics* 15.1, p. 1162. ISSN: 1471-2164. DOI: 10.1186/1471-2164-15-1162.

Schmich, F et al. (Oct. 2015). “gespeR: A Statistical Model for Deconvoluting off-Target-Confounded RNA Interference Screens”. *Genome Biology* 16.1, p. 220. ISSN: 1474-760X. DOI: 10.1186/s13059-015-0783-1.

Schultz, N et al. (2011). “Off-Target Effects Dominate a Large-Scale RNAi Screen for Modulators of the TGF-β Pathway and Reveal microRNA Regulation of TGFBR2.” *Silence*.

Singh, S, AS Narang, and RI Mahato (Dec. 2011). “Subcellular Fate and Off-Target Effects of siRNA, shRNA, and miRNA”. en. *Pharmaceutical Research* 28.12, pp. 2996–3015. ISSN: 1573-904X. DOI: 10.1007/s11095-011-0608-1.

Srivatsa, S et al. (July 2018). “Improved Pathway Reconstruction from RNA Interference Screens by Exploiting Off-Target Effects”. en. *Bioinformatics* 34.13, pp. i519–i527. ISSN: 1367-4803. DOI: 10.1093/bioinformatics/bty240.

Steckel, M et al. (2012). “Determination of Synthetic Lethal Interactions in KRAS Oncogene-Dependent Cancer Cells Reveals Novel Therapeutic Targeting Strategies.” *Cell Research*.

Szklarczyk, D et al. (Jan. 2017). “The STRING Database in 2017: Quality-Controlled Protein–Protein Association Networks, Made Broadly Accessible”. en. *Nucleic Acids Research* 45.D1, pp. D362–D368. ISSN: 0305-1048. DOI: 10.1093/nar/gkw937.

Tang, W et al. (2008). “A Genome-Wide RNAi Screen for Wnt/Beta-Catenin Pathway Components Identifies Unexpected Roles for TCF Transcription Factors in Cancer.” *Proceedings of The National Academy Of Sciences Of The United States Of America*.

Thanei, GA, N Meinshausen, and RD Shah (Jan. 2018). “The Xyz Algorithm for Fast Interaction Search in High-Dimensional Data”. *Journal of Machine Learning Research* 19.1, pp. 1343–1384.

Tiuryn, J and E Szczurek (July 2019). “Learning Signaling Networks from Combinatorial Perturbations by Exploiting siRNA Off-Target Effects”. en. *Bioinformatics* 35.14, pp. i605–i614. ISSN: 1367-4803. DOI: 10.1093/bioinformatics/btz334.

Wright, S (1932). “The Roles of Mutation, Inbreeding, Crossbreeding, and Selection in Evolution”. *Proc 6th Int. Cong. Genet.* 1, pp. 356–366.

Yuan, M and Y Lin (2006). “Model Selection and Estimation in Regression with Grouped Variables”. *J R Stat Soc Series B Stat Methodol.*

Appendix A. Fitness landscapes and epistasis

A.1. Conditional epistasis. For two genes i and j and a fixed set of background perturbations $b = (x_1, \dots, x_{i-1}, x_{i+1}, \dots, x_{j-1}, x_{j+1}, \dots, x_p) \in \mathbb{R}_+^{p-2}$ we define conditional epistasis between gene i and j given b as

$$\varepsilon_{i,j|b} = f(x_1, \dots, x_{i-1}, 0, x_{i+1}, \dots, x_{j-1}, 0, x_{j+1}, \dots, x_n) + f(x_1, \dots, x_{i-1}, 1, x_{i+1}, \dots, x_{j-1}, 1, x_{j+1}, \dots, x_p) - f(x_1, \dots, x_{i-1}, 1, x_{i+1}, \dots, x_{j-1}, 0, x_{j+1}, \dots, x_p) - f(x_1, \dots, x_{i-1}, 0, x_{i+1}, \dots, x_{j-1}, 1, x_{j+1}, \dots, x_p) \tag{11}$$

Proposition 1. *For the fitness landscape model (3), the interaction terms $\beta_{i,j}$ are independent of b and equal to conditional epistasis, that is, $\varepsilon_{i,j|b} = \beta_{i,j}$.*

Proof. Without loss of generality, we can consider $(i, j) = (1, 2)$. Let $b = (x_3, \dots, x_p)$. In model (3) we have

$$\begin{aligned} \varepsilon_{1,2|b} &= f(0,0,x_3,\dots,x_p) + f(1,1,x_3,\dots,x_p) \\ &\quad - f(1,0,x_3,\dots,x_p) - f(0,1,x_3,\dots,x_p) \\ &= \beta_0 + \Big(\beta_0 + \beta_1 + \beta_2 + \beta_{1,2} + \sum_{i>2} x_i\beta_{1,i} + \sum_{i>2} x_i\beta_{2,i}\Big) \\ &\quad - \Big(\beta_0 + \beta_1 + \sum_{i>2} x_i\beta_{1,i}\Big) - \Big(\beta_0 + \beta_2 + \sum_{i>2} x_i\beta_{2,i}\Big) \end{aligned} \tag{12}$$

All terms except the interaction $\beta_{1,2}$ cancel out, therefore $\varepsilon_{1,2|b} = \beta_{1,2}$. □

A.1.1. *Marginal epistasis.* The marginal fitness landscape of genes i and j is

$$f_{i,j}(x_i, x_j) = \sum_{\{x_k \in \{0,1\} | k \neq i,j\}} f(x_1, \dots, x_p) \tag{13}$$

and marginal epistasis between genes i and j is the epistasis of the marginal fitness landscape,

$$\varepsilon_{i,j} = f_{i,j}(0,0) + f_{i,j}(1,1) - f_{i,j}(1,0) - f_{i,j}(0,1) \tag{14}$$

For example, for $p = 3$ genes, marginal epistasis between gene 1 and 2 is

$$\varepsilon_{1,2} = [f(0,0,0) + f(0,0,1)] + [f(1,1,0) + f(1,1,1)] - [f(1,0,0) + f(1,0,1)] - [f(0,1,0) + f(0,1,1)] \quad (15)$$

Note that we consider the marginal epistasis of the *pair* of genes, rather than a single gene with all others as in (Crawford et al. 2017))

Corollary 1. *For the fitness landscape model (3), the interaction terms $\beta_{i,j}$ are related to marginal epistasis via $\varepsilon_{i,j} = 2^{p-2}\,\beta_{i,j}$.* †

Proof. From Proposition 1 we have that conditional epistasis for a pair of genes (i, j) and a fixed genetic background of the remaining $p - 2$ genes equals $\beta_{i,j}$. There are 2^{p-2} such genetic backgrounds, and the conditional epistasis is the same for all of them. □

Thus, in the fitness landscape model (3), which contains all main effects and pairwise interactions, but no interactions of higher order, the interaction terms $\beta_{i,j}$ alone determine conditional and marginal epistasis of the fitness landscape.

Appendix B. Number of epistatic gene pairs

For $n = 10 \times p = 1000$ siRNAs, 87% of the $\binom{p}{2} = 4950$ gene pairs are simultaneously perturbed by at least one siRNA.

† We use this definition of epistasis, rather than defining it in terms of averages, because it preserves $\beta_{i,j}$ as the value actually calculated by our model

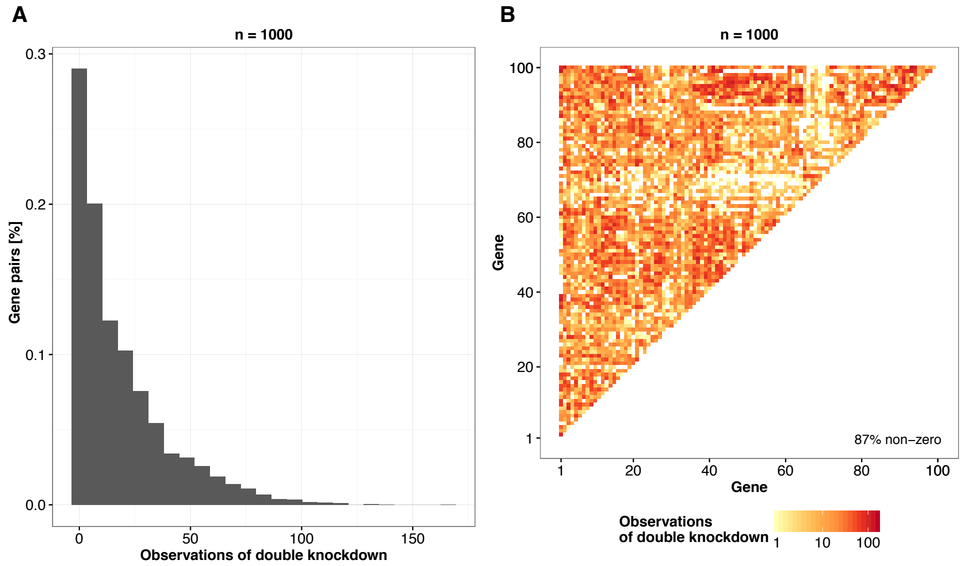

Figure 10. Simulation of perturbation matrices for $n = 1000$ siRNAs and $p = 100$ genes based on four commercial genome-wide siRNA libraries from Qiagen. (**A**) The number of times each pair of genes is simultaneously perturbed in the simulated matrix. (**B**) Heat map of the number of simultaneous perturbations for each gene pair. Darker colour indicates higher numbers of observations. 87% of the $\binom{p}{2}$ pairs are simultaneously perturbed at least once.

An increase in the number of pairs of genes $(i, j) : \beta_{i,j} > 0$, i.e. pairs of genes with true conditional epistasis greater than zero, generally leads to an increase in precision and decrease in recall which results in a subtle increase in F1 when searching with `glinternet` (Figure 11a). The only exception being when there are no additional main effects, in which case interactions are more reliably found from among a small set (between 5 and 20 depending on the SNR) than a large one (50 or more). When we select estimates $\hat{\beta}_{i,j}$ with a magnitude significantly different from zero (Figure 11b), we observe a more than 3-fold increase of precision but steeper decrease of recall for increasing numbers of pairs of genes with true conditional epistasis. This results in approximately 2-fold increase of the F1 measure, which in addition shows a weaker dependency on the number of gene pairs with true conditional epistasis. With an increasing number of additional main effects, the performance generally decreases. The effect is more subtle for high numbers of true epistatic gene pairs, both with and without selecting $\hat{\beta}_{i,j}$ significantly different from zero. As expected, higher SNRs leads to better performance, where this effect is stronger when we perform the significance test. The trade-off between precision and recall resulting from the significance test is shown in Figure 13a. The increase in precision and decrease of recall is stronger for higher number of true epistatic gene pairs. For small numbers of true epistatic gene pairs (5, 20) we observe a dependency of the strength of increase of precision and decrease of recall to the number of additional main effects. Overall, the ratio of increase in precision

and decrease of recall is approximately 3, suggesting that the test in general led to an increase in performance. Figures without this test may be found in Appendix F.

It should be noted that the expected precision of random guessing of interactions is $\frac{q}{p(p-1)}$. This is at most $\approx 1\%$, when $q = 100, p = 100$, as in our simulations.

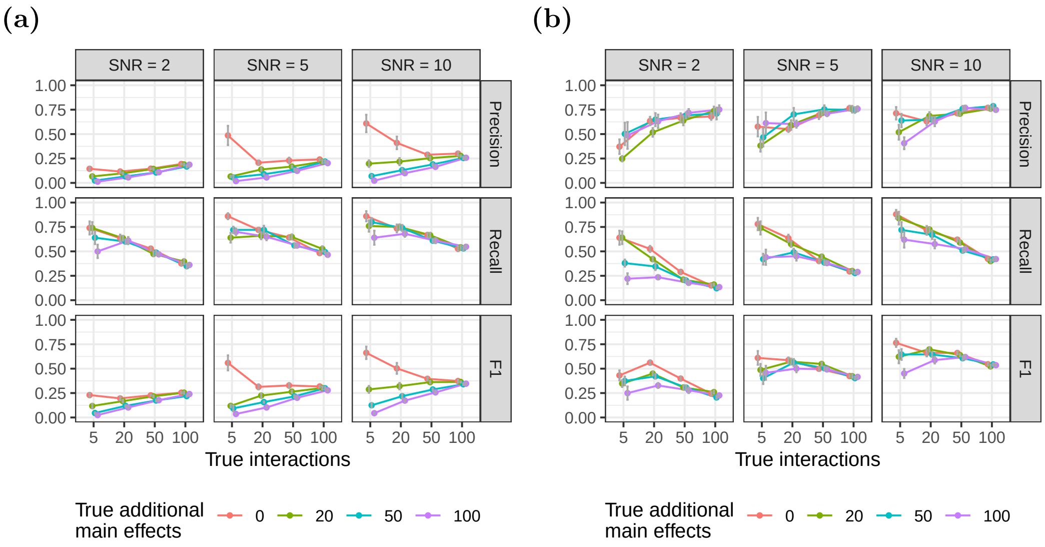

Figure 11. Identification of epistasis for increasing numbers of true interactions using `glinternet`. Panel rows show precision, recall, and the F1 measure and panel columns depict results for signal-to-noise ratios (SNR) 2, 5, and 10. Colour indicates the number of additional main effects not overlapping with the set of interacting genes. (a) Results for all conditional epistasis $\beta_{i,j} > 0$; (b) Results for the subset of conditional epistasis $\beta_{i,j}$ that significantly deviate from zero (q-value < 0.05).

Using the same number of genes and main effects and searching with `xyz`, we see similar precision, albeit with significantly lower recall (Figure 12). As with `glinternet`, performance improved with higher SNRs. Selecting estimates that significantly deviate from zero (q-value < 0.05) results in as much as a 2-fold improvement in precision in the best case, however improvements are generally smaller with `xyz` than with `glinternet`. In this case, the effect on recall is minimal, the trade-off is shown in Figure 13b.

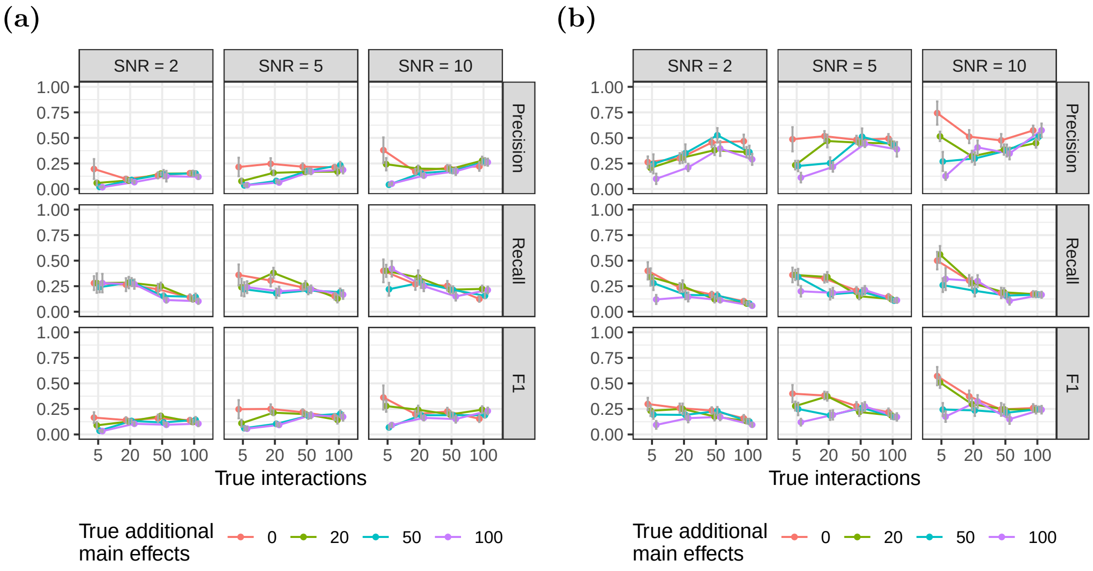

Figure 12. Results for `xyz` as in Figure 11. Note that this format is reused in all such figures. (a) Without significance test. (b) With significance test.

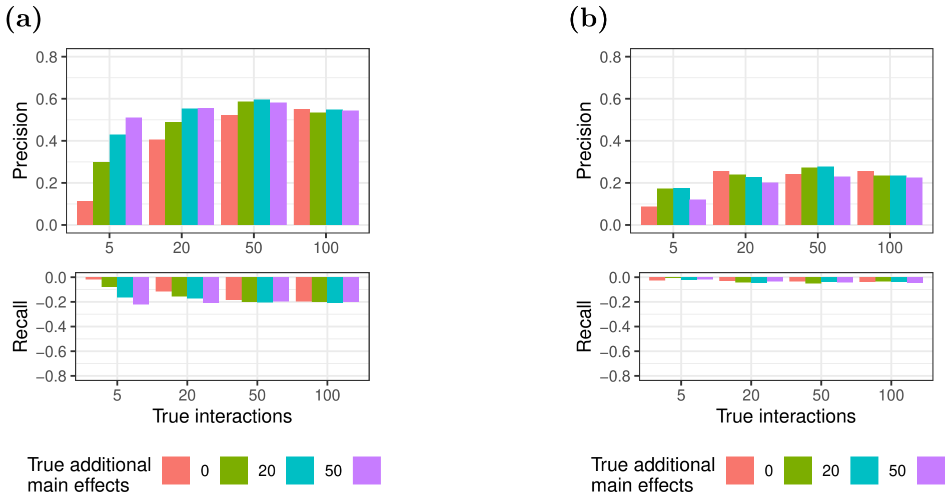

Figure 13. Trade-off between precision and recall for selecting the subset of interactions significantly deviating from zero versus all interactions. Top and bottom panels depict gain of precision and loss of recall, respectively. (a) `glinternet`; (b) `xyz`.

Appendix C. Magnitude

Comparing the estimated magnitude of epistasis to the ground truth, we find the `glinternet` results typically deviate less than 5%, and are only larger with a large number of true interactions, and a low signal to noise ratio. Using `xyz` we can see some significant variation in accuracy. The deviation is, however, typically below 10%.

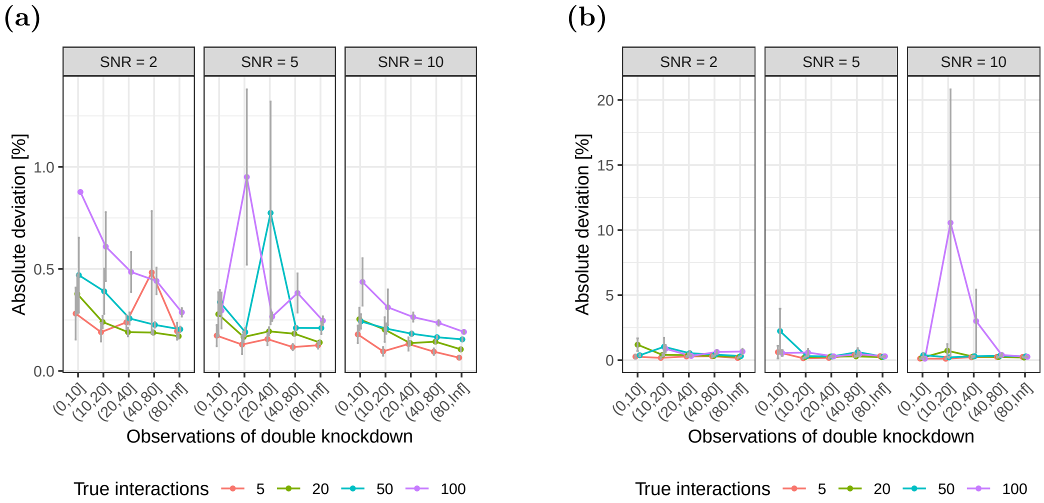

Figure 14. Concordance between the magnitude of true and estimated epistasis. The fraction of incorrectly identified signs between true and estimated epistasis for (a) `glinternet` and (b) `xyz`. Results are for the subset of interactions that significantly deviate from zero (q-value < 0.05).

Appendix D. The `xyz`Algorithm

The xyz algorithm `xyz`defines $\mathbf{Z} \in \mathbb{R}^{n \times p} : Z_{i,j} = Y_i X_{i,j}$, and reduces the problem of finding pairs j, k such that $Y^T(X_j \circ X_k)/n > \lambda$ to that of finding j, k where $||X_j - Z_k||_2 < \lambda'$. The latter is achieved using locality sensitive hashing. Given parameters L, λ, the `xyz`algorithm is as follows:

Algorithm 1 The `xyz`algorithm

Require: L, λ

$I = \emptyset$
for L times **do**
  Choose a random vector $R \in \mathbb{R}^n$, and calculate $x = \mathbf{X}^T\mathbf{R}$ and $z = \mathbf{Z}^T\mathbf{R}$.
  The pairs j, k for which $x_j = z_k$ are candidates for interactions.
  Among these, add the pairs for which $|\mathbf{X}_j^T \mathbf{Z}_k|/n > \lambda$ to the set I.
end for
I is the set of significant Interactions, using the cut-off λ.

The parameter λ here is the same as the linear regression λ, and algorithm 1 can be used during regression to choose the interaction effects worth considering at the current value of λ. Unless otherwise specified, we use the value of $L = \sqrt(n)$. The effect of varying this parameter is considered in Appendix E.

Appendix E. Number of xyz Projections

To ensure the correct xyz parameters are chosen, we compare precision, recall, and F1 for varying numbers of projections. Fixing the signal to noise ration to $SNR = 5$, and using the same parameters as the main $p = 1000$ simulations above, we run xyz with $L = 10, 100$, and 1000.

While there is a clear advantage to running at least $L = \sqrt(n) = 100$ projections, there are no significant gains in overall performance, as indicated by $F1$, beyond that. In fact, we can see in Figure 15c that increasing the number of projections beyond that merely reduces the number of interactions returned, without improving accuracy.

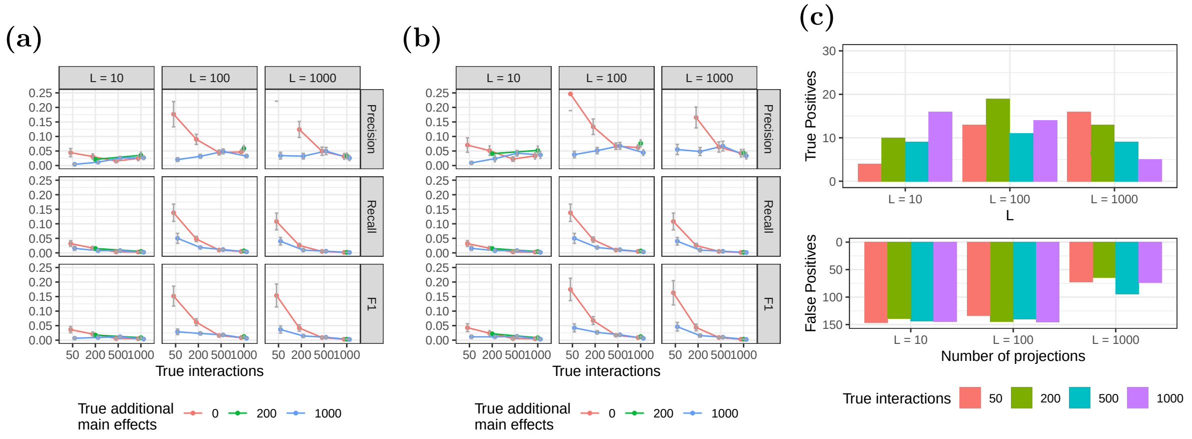

Figure 15. Precision, recall, and F1 as a result of increasing the number of projections. We use $p = 1000$ genes with a signal to noise ratio of five. 15a: Results considering all identified conditional epistasis. 15b: Results considering only the subset of conditional epistasis that significantly deviate from zero. 15c: Number of interactions reported, note that the scale above differs from the one below for readability.

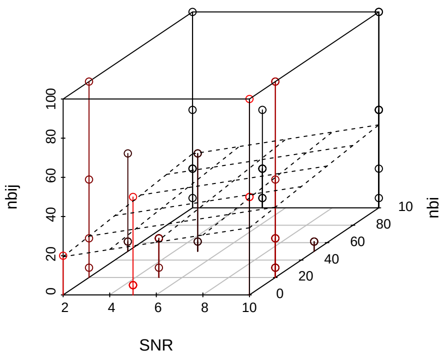

Figure 16. Distribution of `xyz` failures.

Appendix F. Results without Significance Test

The results used above are using R's `lm` linear regression, including only those with significant q-values ($q < 0.05$). Here we perform the same tests with all found effects included, significant or otherwise, or comparison.

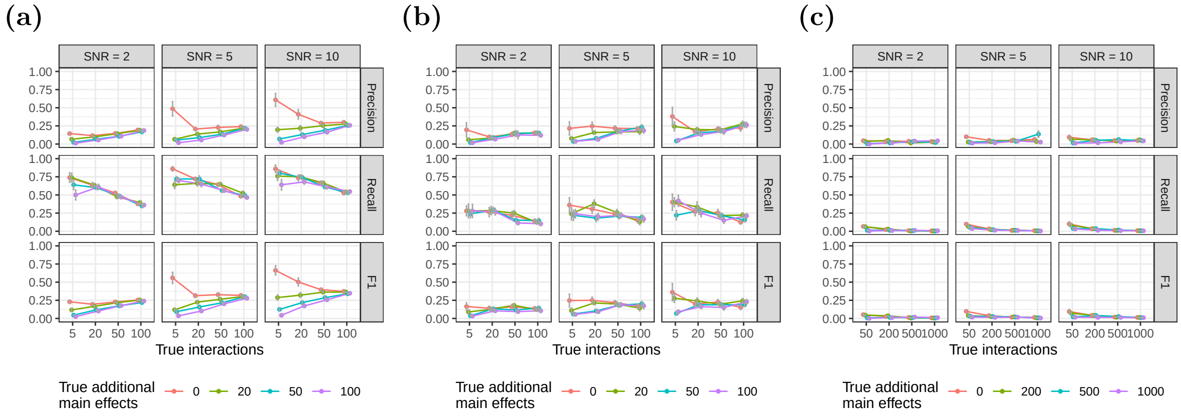

Figure 17. Precision, recall, and F1 performance measures for `xyz`. 17a: Results for all identified epistasis on $p = 100$, $n = 100$ simulation using `glinternet`. 17b: Results for all identified epistasis on $p = 100$, $n = 100$ simulation using `xyz`. 17c: Results for all identified epistasis on $p = 1000$, $n = 10000$ simulation using `xyz`.

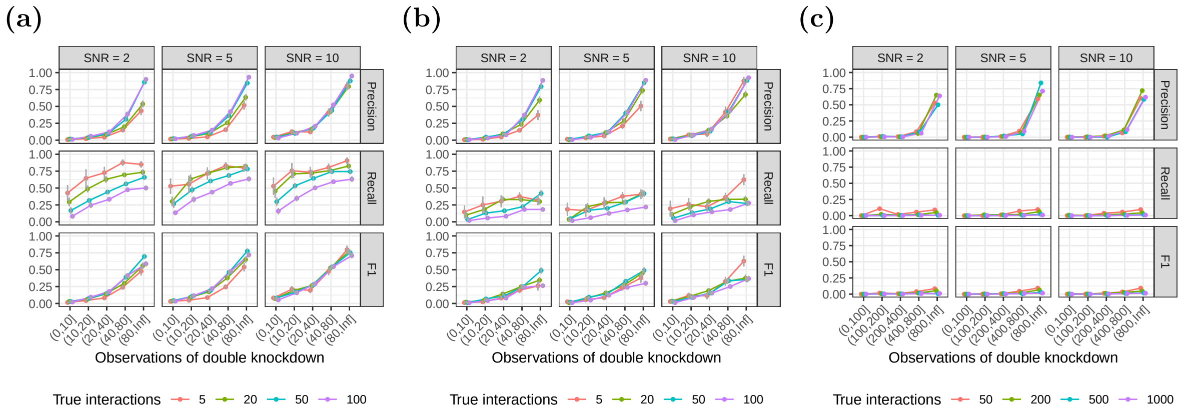

Figure 18. Identification of epistasis for increasing numbers of observations of the pairwise double knockdown. Results are for all identified conditional epistasis $\beta_{i,j} > 0$. (a) Results using `glinternet`. (b) Results using `xyz` on small ($p = 100, n = 1000$) simulations. (c) Results using `xyz` on large ($p = 1000, n = 10000$) simulations.

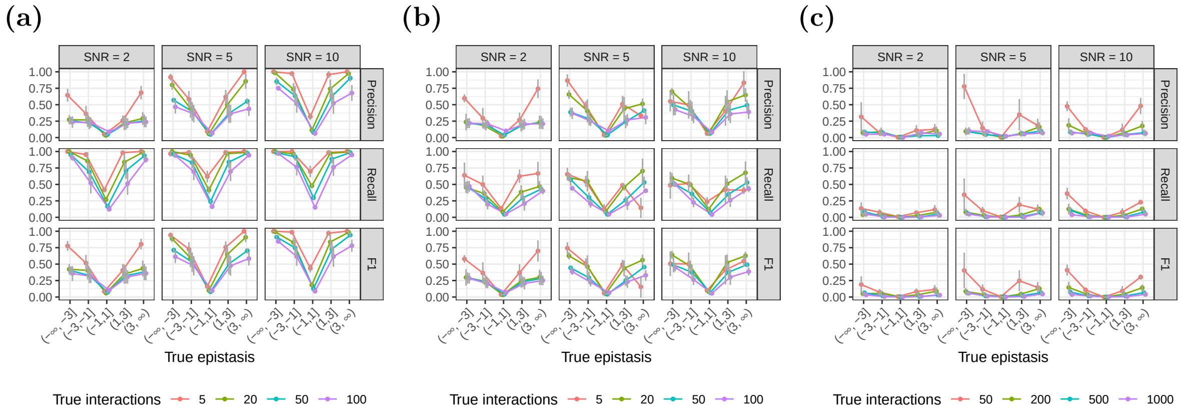

Figure 19. Identification of epistasis for varying effect size. Results are for all identified conditional epistasis $\beta_{i,j} > 0$. (b) Small ($p = 100, n = 1000$) simulations, using `glinternet`. (b) Small ($p = 100, n = 1000$) simulations, using `xyz`. (c) Large $p = 1000,\ n = 10000$ simulations, using `xyz`.

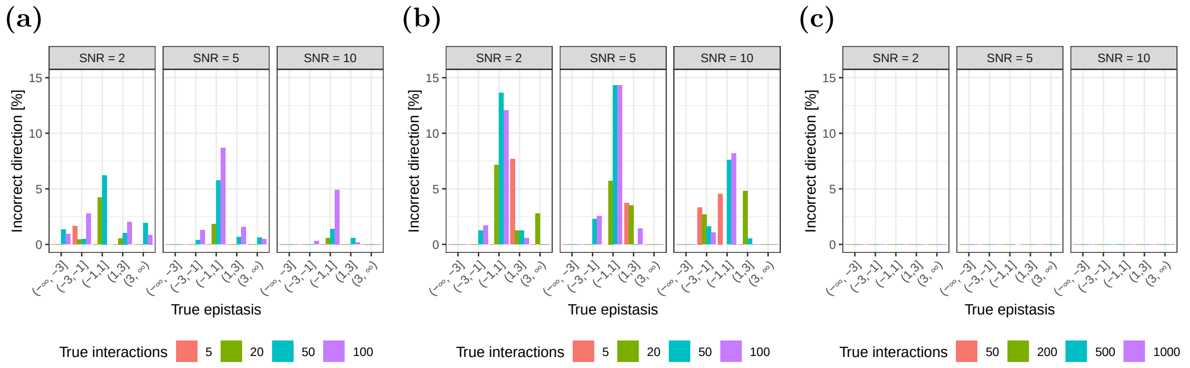

Figure 20. Identification of epistasis for varying effect size. Results are for all identified conditional epistasis $\beta_{i,j} > 0$. (b) Small $(p = 100, n = 1000)$ simulations, using `glinternet`. (b) Small $(p = 100, n = 1000)$ simulations, using `xyz`. (c) Large $p = 1000,\ n = 10000$ simulations, using `xyz`. Note that in this test there are no incorrect results

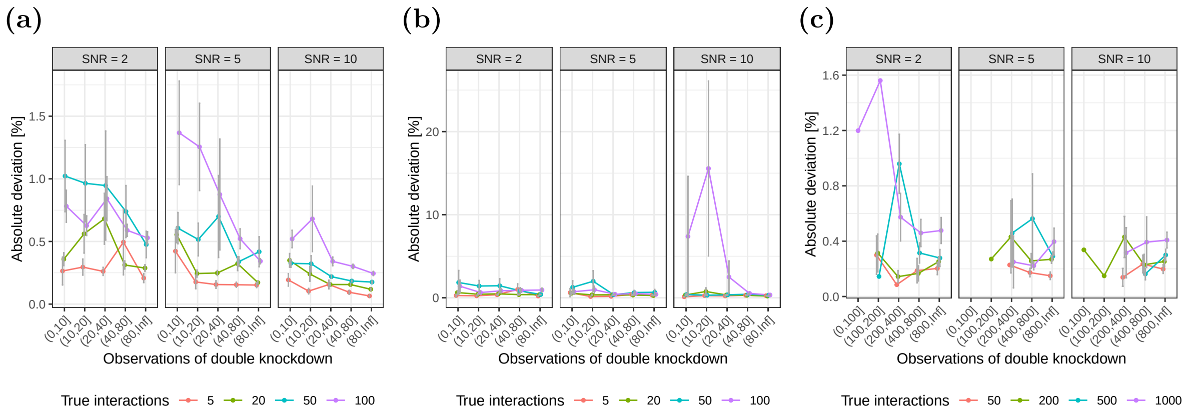

Figure 21. Concordance between the magnitude of true and estimated epistasis. Results are for all identified conditional epistasis $\beta_{i,j} > 0$. (a) Small $(p = 100, n = 1000)$ simulations, using `glinternet`. (b) Small $(p = 100, n = 1000)$ simulations, using `xyz`. (c) Large $p = 1000,\ n = 10000$ simulations, using `xyz`.

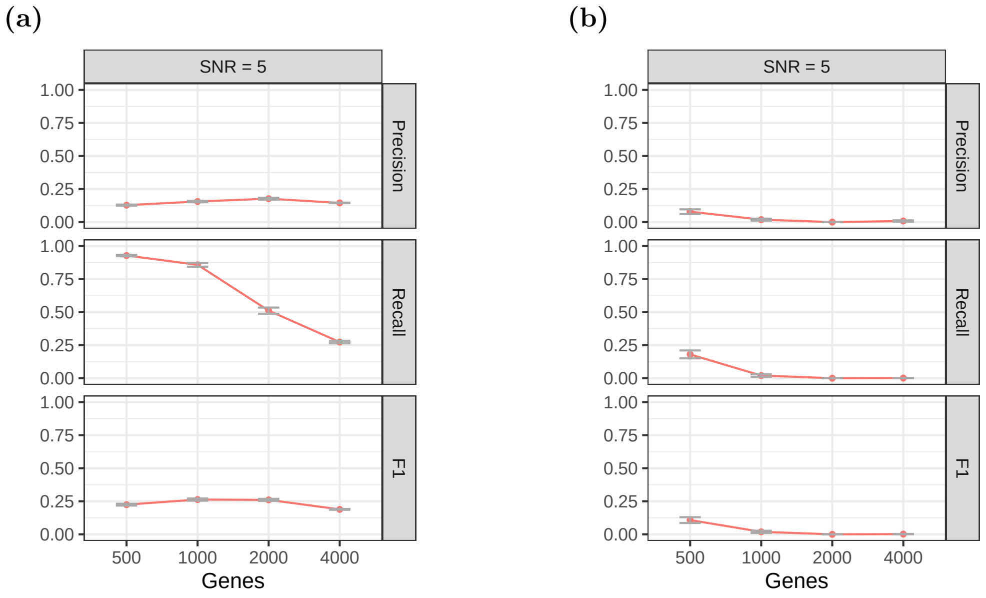

Figure 22. The performance of (a) `glinternet` and (b) `xyz` on increasingly large data sets. Results are for all identified conditional epistasis $\beta_{i,j} > 0$.
