# Supplementary material for: Learning epistatic gene interactions from perturbation screens
Source: PLoS One. 2021 Jul 13;16(7):e0254491. doi: 10.1371/journal.pone.0254491 (PMC8277066; doi:10.1371/journal.pone.0254491)
Supplement: Attachment — Submitted filename: response to reviewers1.pdf [file pone.0254491.s003.pdf]

Response to PLOS One Comments

May 28, 2021

Dear Prof Artero,

We would like to thank you and the referees for the helpful feedback. We have addressed all the comments and provide a detailed response below.

1 Reviewer #1

Reviewer #1: This paper describes a technique for inferring epistatic interactions from siRNA perturbation screens. The main ideas are (1) to treat the off-target effects of each siRNA together with the on-target effects as a complex perturbation of many genes and then (2) to extract epistatic interactions from a collection of the complex perturbations using a sparse regression framework. The paper sets up this framework, conducts an extensive simulation study where this approach is implemented using two different existing methods for sparse (elastic net) regression, and provides a reanalysis of an existing siRNA dataset.

The contribution here is sound, and indeed a rather creative idea, and is certainly suitable for PLoSOne with **a few minor corrections**.

1. The definition of marginal epistasis could be improved by **defining it in terms of averages rather than a sums.** Then the fitness of a genotype in the marginal fitness landscape would simply be the expected fitness of a genotype given its allelic state at the specified subset of genes, and Corollary 1 simplifies to $\epsilon_{i,j} = \beta_{i,j}$ instead of $\epsilon_{i,j} = 2^{p-2}\beta_{i,j}$.

    - This simplifies the definition of epistasis, but the β_{i_j} values given are the parameters our model actually estimates. This is purely to show that this is equivalent to estimating epistasis. We have clarified this point in a footnote on page 25.

2. The authors **should also disambiguate** between their concept of marginal epistasis and the (different) marginal epistasis concept studied in e.g. Crawford et al. 2017 PLoS Genetics.

    - This has been clarified in the text on page 23, Appendix A.

3. Sparse regression (Lasso, elastic map, and their Bayesian analogs) has previously been applied both in the fitness landscape and the quantitative genetics literature, and **the reader should be aware of how the current contribution fits into this broader context**. I am thinking of things like Cai et al. 2011 "Fast empirical Bayesian LASSO for multiple

quantitative trait locus mapping" in the quantitative genetics literature and Otwinowski and Nemenman 2013 and Poelwijk et al. 2019 in the fitness landscape literature, but there is additional work in both areas (especially in quantitative genetics). One important feature of L1 regularized regression is that the **results are sensitive to the choice of basis,** and the **Taylor basis used here is one difference** with most other treatments of epistasis via regularized regression.

- Poelwijk et al. 2019 and Nemenman 2013 study near complete measurements of very small genotype spaces (on the order of dozens of genes). This is important for understanding epistasis in general, but inapplicable to human genome scale data. We have added these to the discussion of smaller-scale fitness landscapes in the introduction.
- It has also been clarified on page four that the Taylor approximation results in the same definition of epistasis as Nemenman 2013.
- The Bayesian Lasso method from Cai et al. 2011 improves accuracy over glmnet, but not running time. Glmnet is already far too slow for human genome-scale interactions, so this method is also not suitable for our purposes.

4. The re-analysis of the siRNA screen should be clarified. The current text says that the kinases being targeted are kinases of the pathogens, but it appears **that these are in fact the kinases of the human host.** More generally, the reader needs more background on the experimental design of this study in order to understand the reanalysis. In Table 1, the **reader also needs to see the corresponding single mutation effects** in order to understand what kind of epistatic interactions these are (e.g. which of these are synthetic lethals), and some of the estimated effects seem awfully large (e.g. 172.04 on a log 2 scale).

- This has been corrected in section 2.6.
- Main effects have been included in Table 1 for comparison.

1.1 Minor additional comments:

1. More precise notation would be helpful in Eqn 6

- This has been expanded.

2. pg. 6 Karush-Kuhn-Tucker conditions

> - These have now been defined in equation 10.

3. pg. 7 θ needs to be defined

> - This has been defined after equation 8.
> - Equations 7-8 should also have referred to interactions between non-identical columns, (i.e. excluding $X_1 \circ X_1$). This has also been corrected.

4. pg. 17 What does failed to run mean?

> - This has been changed to 'produced no results'.

2 Reviewer #2

1. I have concerns about the two-stage approach to identify interactions. Using linear model to calculate estimates and p values does not take in account the selection process of LASSO. Refitting the regression model after variable selection may lead to biased estimates. However, some advances have been done in this area (Taylor & Tibshirani, 2016; D. Lee, J. et al, 2016).

> - The current implementations of these methods require calculating p-values using the complete X' interaction matrix, which is in general too large to work with. These calculations can therefore only be done from within the glinternet or xyz packages (where the matrix is internally represented) and adding this to either method is beyond the scope of our work.
> - A paragraph explaining why we cannot use current implementations of these approaches, and noting the potential for biased estimates, has been added to the discussion, and we now mention at the end of section 1.2.3 that these estimates may be biased.

2. Importance of interactions is selected based on p value, however the criteria should be effect size along with p value. Consequently, please, review the following sentence from page 8 "We are nonetheless able (...)" I would suggest to remove it.

- This has been removed.

3. I would like the authors to explain if the impact of the scalability could be a problem in real world data.

- A brief discussion of this has been added to the scalability section.

4. As a general comment, strong emphasis is done to simulated data but there is a need to further investigate the effects of this methodology in real world data.

- A paragraph on real-world verification has been added to the discussion, but we must stress that experimental verification of the results is beyond the scope of our work.

5. The last paragraph of the results section compares R2 of two models, however it is well-known that R2 increases when including more covariates. It is recommended to compare models with a complexity penalization approach, i.e, AIC.

- This has been changed in the text at the end of section 2.6, the discussion now refers to the improved AIC.

6. I would like to know if the authors studied the role of epistatic gene interactions in any specific disease. The presence of some clinical example could improve the quality of the article making it more easily readable.

- We have not studied any particular diseases, and this would be a good application of our method. Doing so would require thoroughly investigating the proposed interactions in a particular data set, and understanding their mechanism. This is well beyond the scope of our work, however. We have added this to our suggestions for future work in the discussion.

7. As a minor comment, when referring to the Normal distribution in an equation it is conventionally assumed to write it as N(,) instead of Norm(,)

- This has been corrected.

3 PLOS ONE Requirements

Please include the following as our funding statement.

3.1 Finding Statement

This work has partially been funded by SystemsX.ch, the Swiss Initiative in Systems Biology, under IPhD grant 2009/025 and RTD grants 51RT-0_126008 (InfectX) and 51RTP0_151029 (TargetInfectX), evaluated by the Swiss National Science Foundation.

AG and KE acknowledge support from the Royal Society Te Apārangi through a Rutherford Discovery Fellowship (RDF-UOO1702) awarded to AG. AG and KE were partially supported by Ministry of Business, Innovation, and Employment of New Zealand through an Endeavour Smart Ideas grant (UOOX1912) and a Data Science Programmes grant (UOAX1932).

JJ is an employee of Novartis Institutes for BioMedical Research, which provided support in the form of the salary, but did not have any additional role in the study design, data collection and analysis, decision to publish, or preparation of the manuscript. This does not alter our adherence to PLOS ONE policies on sharing data and materials.

3.2 List of changes

- ☑ A rebuttal letter that responds to each point raised by the academic editor and reviewer(s). You should upload this letter as a separate file labeled ‘Response to Reviewers’.
- ☑ A marked-up copy of your manuscript that highlights changes made to the original version. You should upload this as a separate file labeled ‘Revised Manuscript with Track Changes’.
- ☑ An unmarked version of your revised paper without tracked changes. You should upload this as a separate file labeled ‘Manuscript’.
- ☑ Please ensure that your manuscript meets PLOS ONE’s style requirements, including those for file naming.
- ☑ We note that you have provided funding information that is not currently declared in your Funding Statement. However, funding information should not appear in the Acknowledgments section or other areas of your manuscript. We will only publish funding information present in the Funding Statement section of the online submission form. Please remove any funding-related text from the manuscript and let us know how you would like to update your Funding Statement. Currently, your Funding Statement reads as follows: [Funded: The required information is available in the Acknowledgements section of the manuscript.]
- ☑ Please include your amended statements within your cover letter; we will change the online submission form on your behalf.
- ☑ Please also include the following statement within your amended Funding Statement. “The funder provided support in the form of salaries for authors [insert relevant initials], but did not have any additional role in the study design, data collection and analysis, decision to publish, or preparation of the manuscript. The specific roles of these authors are articulated

in the ‘author contributions’ section.” If your commercial affiliation did play a role in your study, please state and explain this role within your updated Funding Statement.

- ☑ Please also provide an updated Competing Interests Statement declaring this commercial affiliation along with any other relevant declarations relating to employment, consultancy, patents, products in development, or marketed products, etc. Within your Competing Interests Statement, please confirm that this commercial affiliation does not alter your adherence to all PLOS ONE policies on sharing data and materials by including the following statement: “This does not alter our adherence to PLOS ONE policies on sharing data and materials.” (as detailed online in our guide for authors http://journals.plos.org/plosone/s/competing-interests) . If this adherence statement is not accurate and there are restrictions on sharing of data and/or materials, please state these. Please note that we cannot proceed with consideration of your article until this information has been declared. Please include both an updated Funding Statement and Competing Interests Statement in your cover letter. We will change the online submission form on your behalf.

- ☑ Please remove your figures from within your manuscript file, leaving only the individual TIFF/EPS image files, uploaded separately. These will be automatically included in the reviewers’ PDF.

- ☑ Please ensure that you refer to Figure 14, 17, 18, 19, 20, 21 and 22 in your text as, if accepted, production will need this reference to link the reader to the figure.
